# Human neural stem cell-derived extracellular vesicles protect against Parkinson’s disease pathologies

**DOI:** 10.1186/s12951-022-01356-2

**Published:** 2022-04-25

**Authors:** Eun Ji Lee, Yoori Choi, Hong J. Lee, Do Won Hwang, Dong Soo Lee

**Affiliations:** 1 Department of Nuclear Medicine, Seoul National University College of Medicine, 101 Daehak-ro, Jongno-gu, Seoul, 03080 South Korea; 2 Department of Nuclear Medicine, Seoul National University Hospital, Seoul, South Korea; 3 Department of Molecular Medicine and Biopharmaceutical Sciences, Graduate School of Convergence Science and Technology, Seoul National University, Seoul, South Korea; 4 College of Medicine and Medical Research Institute, Chungbuk National University, Cheongju, South Korea; 5 Research Institute, huMetaCELL Inc., 220, Bugwang-ro, Puchon, Gyeonggi-do Republic of Korea; 6 THERABEST, Inc., Seocho-daero 40-gil 41, Seoul, 06656 South Korea

**Keywords:** Extracellular vesicles, Neural stem cell, Parkinson’s disease, Oxidative stress, Neuroinflammation

## Abstract

**Background:**

Neural stem cells (NSCs) have the ability to generate a variety of functional neural cell types and have a high potential for neuronal cell regeneration and recovery. Thus, they been recognized as the best source of cell therapy for neurodegenerative diseases, such as Parkinson’s disease (PD). Owing to the possibility of paracrine effect-based therapeutic mechanisms and easier clinical accessibility, extracellular vesicles (EVs), which possess very similar bio-functional components from their cellular origin, have emerged as potential alternatives in regenerative medicine.

**Material and methods:**

EVs were isolated from human fibroblast (HFF) and human NSC (F3 cells). The supernatant of the cells was concentrated by a tangential flow filtration (TFF) system. Then, the final EVs were isolated using a total EV isolation kit.

**Results:**

In this study, we demonstrate the potential protective effect of human NSC-derived EVs, showing the prevention of PD pathologies in 6-hydroxydopamine (6-OHDA)-induced in vitro and in vivo mouse models. Human NSC and F3 cell (F3)-derived EVs reduced the intracellular reactive oxygen species (ROS) and associated apoptotic pathways. In addition, F3-derived EVs induced downregulation of pro-inflammatory factors and significantly decreased 6-OHDA-induced dopaminergic neuronal loss in vivo. F3 specific microRNAs (miRNAs) such as hsa-mir-182-5p, hsa-mir-183-5p, hsa-mir-9, and hsa-let-7, which are involved in cell differentiation, neurotrophic function, and immune modulation, were found in F3-derived EVs.

**Conclusions:**

We report that human NSC-derived EVs show an effective neuroprotective property in an in vitro transwell system and in a PD model. The EVs clearly decreased ROS and pro-inflammatory cytokines. Taken together, these results indicate that NSC-derived EVs could potentially help prevent the neuropathology and progression of PD.

**Graphical Abstract:**

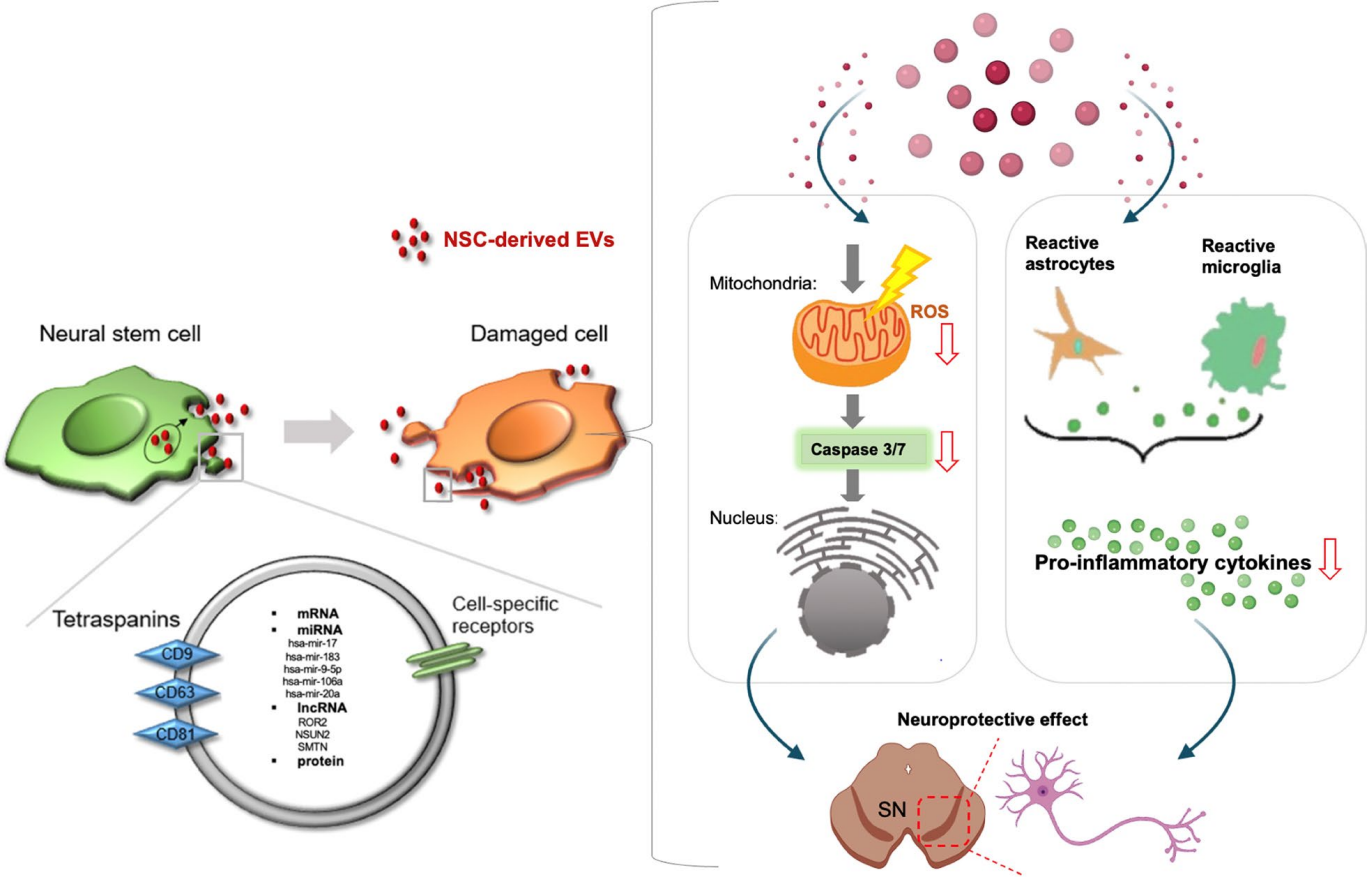

**Supplementary Information:**

The online version contains supplementary material available at 10.1186/s12951-022-01356-2.

## Background

The main pathological feature of Parkinson’s disease (PD) is the progressive loss of dopaminergic neurons in the substantia nigra (SN), accompanied by a severe deficiency of dopamine concentration in the striatum [1]. Although the cause of dopaminergic neuronal degeneration is unknown, the involvement of mitochondrial dysfunction, neuroinflammation, protein clearance defects, and α-synuclein accumulation have been reported in postmortem examinations, brain imaging, and animal studies [2, 3].

Mitochondrial dysfunction contribute to the degeneration of dopaminergic neurons in PD pathogenesis. Excessive reactive oxygen species (ROS), which induce oxidative stress on lipids, proteins, and nucleic acids [4], damage the mitochondria and other cellular structures to trigger apoptosis. 6-hydroxydopamine (6-OHDA), which is taken up into cells via the dopamine reuptake transporter in dopaminergic neurons, has been used to generate a state of oxidative stress in PD. The 6-OHDA-induced PD models lead to the loss of dopaminergic neurons, which is caused by ROS-mediated oxidative stress [5].

Neuroinflammation is a common feature of PD pathology [6]. In patients with PD, increased microglial reactivity, as observed using brain positron emission tomography (PET) imaging, and pro-inflammatory cytokines in plasma and cerebrospinal fluid have been reported [7, 8]. This neuroinflammation in PD is accompanied by changes in microglia, astrocytes, and infiltration of immune cells [9]. In particular, the increase and over-activation of T helper 17 (Th17) cells in the brain tissue of patients with PD was observed as one of the main causes of chronic inflammation [10]. The infiltrated peripheral immune cells and reactive glial cells release pro-inflammatory cytokines and chemokines, which can exert neurotoxic effects by disrupting the normal neuroprotective actions of glial cells. These results indicate the possibility of regulating the progression of PD through the modulation of reactive glial cells and protect neurons in the PD model [11].

Current PD treatments provide symptomatic relief but do not reverse disease progression. Gene therapy and cell therapy have recently been applied as treatment options for PD [12]. Gene therapy strategies include modifications of neural circuits, such as gamma-aminobutyric acid basal tone, dopamine synthesis, and growth factor support [13]. In addition, as a cell therapy strategy, transplantation approaches of dopaminergic neurons derived from a variety of stem cells have been proposed. Neural stem cells (NSCs) have shown potential therapeutic effects by suppressing inflammation and inducing neuronal regeneration in damaged brain cells [14]. These effects are known to be involved in a paracrine signaling-based biological mechanism which rescues the lost neuronal population and improves cell function [15].

Although these cell-based strategies are suitable for supporting neurons, they still have limitations such as safety, immunogenicity, and delivery efficiency. As an alternative therapy, extracellular vesicles (EVs), which play a key role in intercellular communication through the transmission of unique molecular signatures, have been proposed [16]. Stem cell-derived EVs are from cells of origin that can regulate tissue regeneration [17, 18]. In particular, hypothalamic NSC-derived exosomes significantly slow senescence-mediated NSC loss through exosomal miRNA delivery [19].

In this study, we verified the protective effect of human NSC, F3 cell (F3)-derived EVs in dopaminergic neurons against 6-OHDA-mediated neurotoxicity. And we investigated the upregulated miRNAs inside F3-derived EVs, which could explain the protective mechanism through neuronal differentiation and neurotrophic function.

## Results

### Protective effect of F3 cells on 6-OHDA treated SH-SY5Y cells

The 6-OHDA toxin is frequently used to induce PD-like effects in both in vitro and in vivo models. We tested the toxic effects of 6-OHDA on SH-SY5Y cells. 6-OHDA treatment significantly decreased the cell viability. The survival rate of SH-SY5Y cells was approximately 50% when the cells were treated with 450 µM 6-OHDA for 24 h, as compared with that of the vesicle-treated control cells (Additional file 1: Fig. S1A).

To test whether F3 cells indirectly showed a positive effect on damaged SH-SY5Y cells, we adopted a transwell system mimicking an in vitro PD environment. The 0.4-µm transwell membrane pore size allowed passage of exosomes, but not of larger microvesicles. Compared to the 6-OHDA only treated group, co-culturing of F3 cells protected degenerating SH-SY5Y cells with 6-OHDA neurotoxin, showing an increase in the ratio of living cells to dead cells (Additional file 1: Fig. S1B).

### Anti-oxidative stress effect of F3-derived EVs on 6-OHDA-induced SH-SY5Y cells

**Fig. 1:**
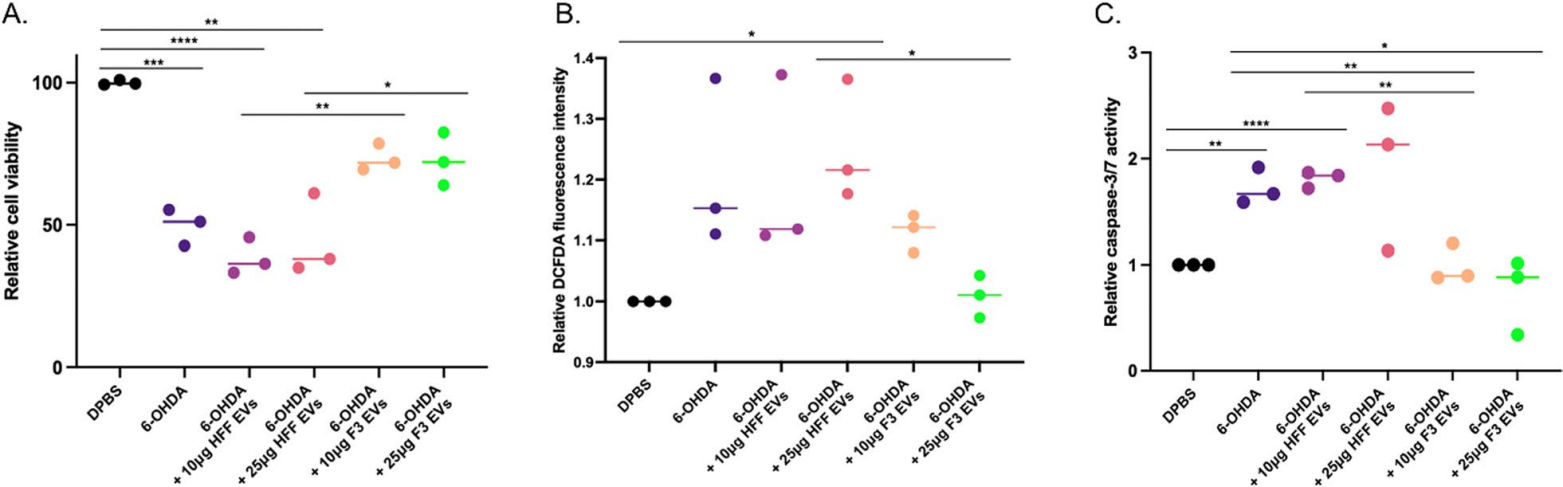
Antioxidant effect of F3-derived EVs on 6-OHDA-induced oxidative stress in SH-SY5Y cells. SH-SY5Y (human neuroblastoma cell lines used as a model of dopaminergic neurons) exposed to 6-OHDA (450 µM) were treated with HFF-derived EVs (10 or 25 µg) or F3-derived EVs (10 or 25 µg). **A** Cell viability was measured using the CCK-8 assay 24 h after EV treatment. 6-OHDA-induced SH-SY5Y cell death was significantly reduced by treatment with F3-derived EVs. **B** Cellular ROS levels were measured using DCFDA. F3-derived EVs significantly reduced 6-OHDA-induced oxidative stress in SH-SY5Y cells. **C** Caspase 3/7 activity was measured using a luminescence assay. Caspase 3/7 activity was reduced in 6-OHDA-induced SH-SY5Y cells treated with F3-derived EVs. *p < 0.05, **p < 0.01, ***p < 0.001, ****p < 0.0001 (one-way ANOVA)

We investigated whether EVs secreted from F3 cells can protect SH-SY5Y cells from 6-OHDA-induced damage. Prior to treatment, EVs derived from HFF and F3 cells were isolated and the markers and size of EVs validated. We identified markers in different categories of EV proteins [20]. Calnexin, an intracellular protein marker, was only found in cell lysate samples, whereas membrane and cytoplasmic proteins were present in EVs (Additional file 1: Fig. S2A). Also, as a result of measuring the size of EVs, it was around 80–150 nm. Both cells identified 5.8 × 10^8^ particles per mL in 10 µg of EV solution (Additional file 1: Fig. S2B). Treatment of 10 or 25 µg of purified EVs from F3 cells significantly attenuated 6-OHDA-induced SH-SY5Y cell toxicity, compared to EVs from HFF cells (Fig. 1A).

It has been reported that 6-OHDA-induced neuronal apoptosis is mediated by intracellular ROS formation due to mitochondrial dysfunction [4]. 6-OHDA-induced SH-SY5Y cells exhibited increased ROS generation in a dose-dependent manner (Additional file 1: Fig. S3A). Interestingly, the ROS generation was significantly reduced in cells treated with 25 µg of F3-derived EVs (Fig. 1B). In addition, 6-OHDA treatment caused a reduction in the MMP with mitochondrial depolarization (Additional file 1: Fig. S3B). The treatment of F3-derived EVs inhibited mitochondrial dysfunction compared to HFF-derived EV-treated group (Additional file 1: Fig. S3E).

While ROS accumulation is known to activate the pro-apoptotic caspase-3/7, we observed an increase in caspase-3/7 activity in 6-OHDA treated SH-SY5Y cells in a dose-dependent manner (Additional file 1: Fig. S3C). An approximately two-fold increase in caspase-3/7 activity was observed with 6-OHDA exposure compared to that in the control group. However, caspase-3/7 activity was significantly suppressed by 10 µg and 25 µg of F3-derived EVs (Fig. 1C). These results revealed that F3-derived EVs decreased the 6-OHDA-mediated induction of apoptosis. Next, we evaluated whether F3-derived EVs prevented 6-OHDA-induced apoptosis of SH-SY5Y cells. The population of PI-stained late apoptotic cells induced by 6-OHDA was gradually increased. Treatment with 6-OHDA increased the percentage of PI-positive cells to 49.2% (Additional file 1: Fig. S3D). In contrast, the toxin-induced increase in late apoptotic cell population was prevented by F3-derived EV treatment at a concentration of 25 µg, compared with that seen in 6-OHDA-treated cells (Additional file 1: Fig. S3F).

### Anti-inflammatory effect of F3-derived EVs on increased pro-inflammatory cytokine levels of BV2 cells by 6-OHDA treated SH-SY5Y cells

**Fig. 2:**
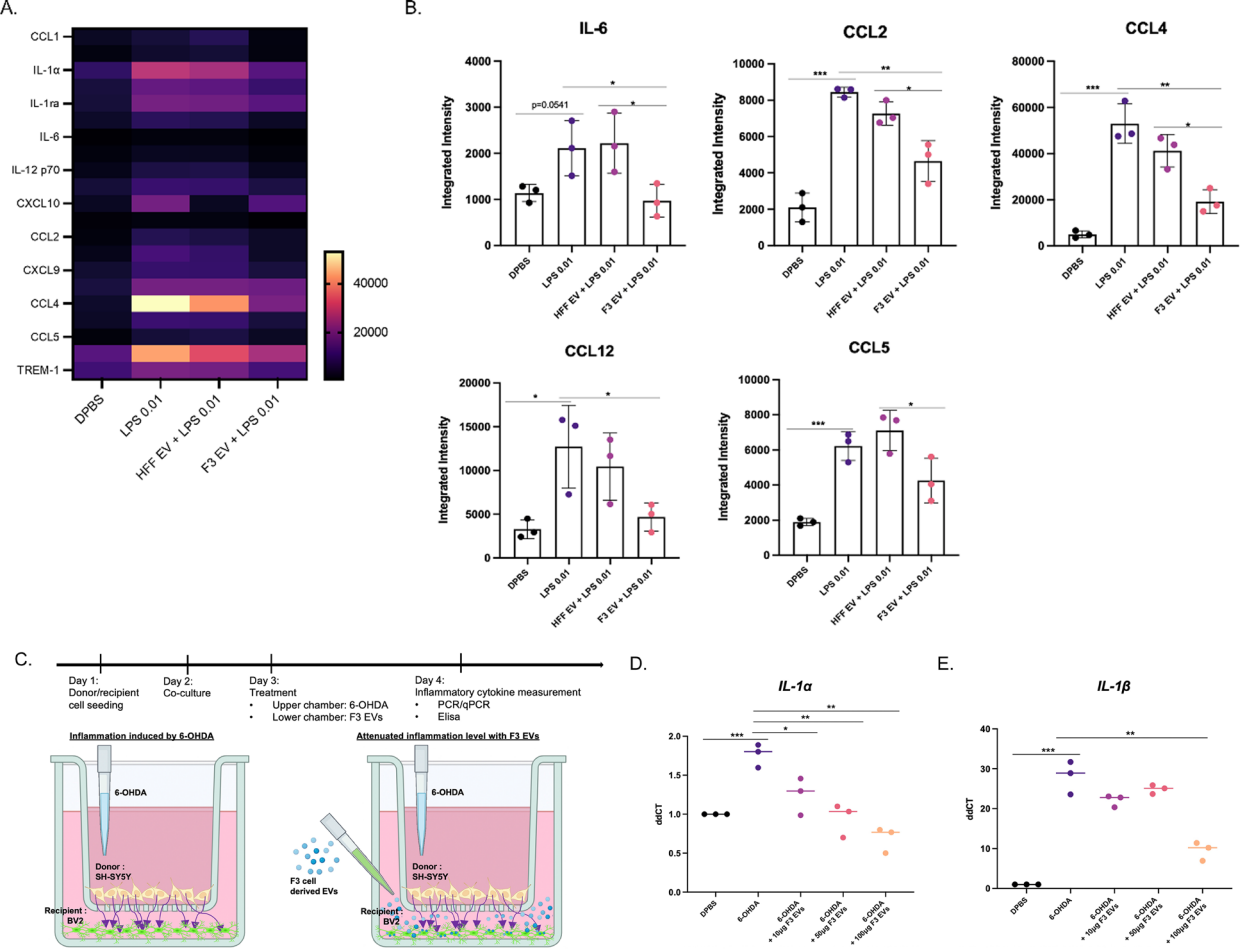
Anti-inflammatory effect of F3-derived EVs on LPS-treated BV2 cells and 6-OHDA-treated SH-SY5Y cells. **A** A heatmap of cytokine and chemokine levels in each group was shown. The changed levels were indicated in colors (yellow-increased, Purple-decreased). (Group: DPBS, LPS 0.01 µg/mL treated, 25 µg of HFF-EV treated with LPS, 25 µg of F3-EV treated with LPS). **B** Five significantly decreased integrated intensity of each cytokine and chemokines in the F3 EV + LPS group compared to LPS and HFF EV + LPS groups were identified. **C** Schematic illustration of the experimental design to validate the change of pro-inflammatory cytokine in BV2 (microglia cell line) co-cultured with 6-OHDA-treated SH-SY5Y cells using the transwell system. **D**, **E** After 24 h of treatment with F3-derived EVs to BV2 (bottom well), the expression of *IL-1α* and *IL-1β* of BV2 cells were measured using qPCR. Intracellular *IL-1α* and *IL-1β* levels were increased in BV2 cells cultured with 6-OHDA-treated SH-SY5Y cells in transwell, but the expression of these cytokines was not increased by F3-derived EVs

As neuroinflammation is another pathological change in PD induced by activated microglia [9], we examined whether F3-derived EVs change inflammatory cytokine levels in BV2, a microglial cell line. LPS treatment induced an increase in pro-inflammatory cytokines in BV2 cells (Additional file 1: Fig. S4A, B). Treatment with F3-derived EVs resulted in a decrease in intracellular interleukin-1 alpha (*IL-1α*) and interleukin-1 beta (*IL-1β*) compared to HFF-derived EVs (Additional file 1: Fig. S4C–E). In addition, while the level of tumor necrosis factor-alpha (TNF-α) dramatically increased after LPS treatment, the level of TNF-α was significantly attenuated after treatment with F3-derived EVs (Additional file 1: Fig. S4F). Then, cytokine arrays were performed to compare changes in cytokines and chemokines at the protein level of microglia between groups. The result showed increased cytokines (IL-1a, IL-1ra, IL-6, IL-12 p70) and chemokines (CCL1, CXCL10, CCL2, CXCL9, CCL4, CCL5) after LPS treatment compared to DPBS treated group (Fig. 2A). Groups treated with F3 EVs (25 µg) showed reduced tendencies of both cytokine and chemokine compared to groups treated with LPS alone and HFF EV (25 µg). We selected five cytokine and chemokines (IL-6, CCL2, CCL4, CCL12, CCL5) that were significantly reduced after the treatment of F3 EVs (Fig. 2B).

To verify whether 6-OHDA induces neuroinflammation in microglia located nearby, 6-OHDA-treated SH-SY5Y cells were co-cultured with BV2 cells. F3-derived EVs were added to the lower bottom chamber seeded with BV2 cells (Fig. 2C). While 6-OHDA treatment increased intracellular *IL-1α* and *IL-1β* levels, these levels were significantly attenuated after administration of F3-derived EVs in a dose-dependent manner (Fig. 2D, E).

### Small RNA expression profiling of F3- and HFF-derived EVs

**Fig. 3:**
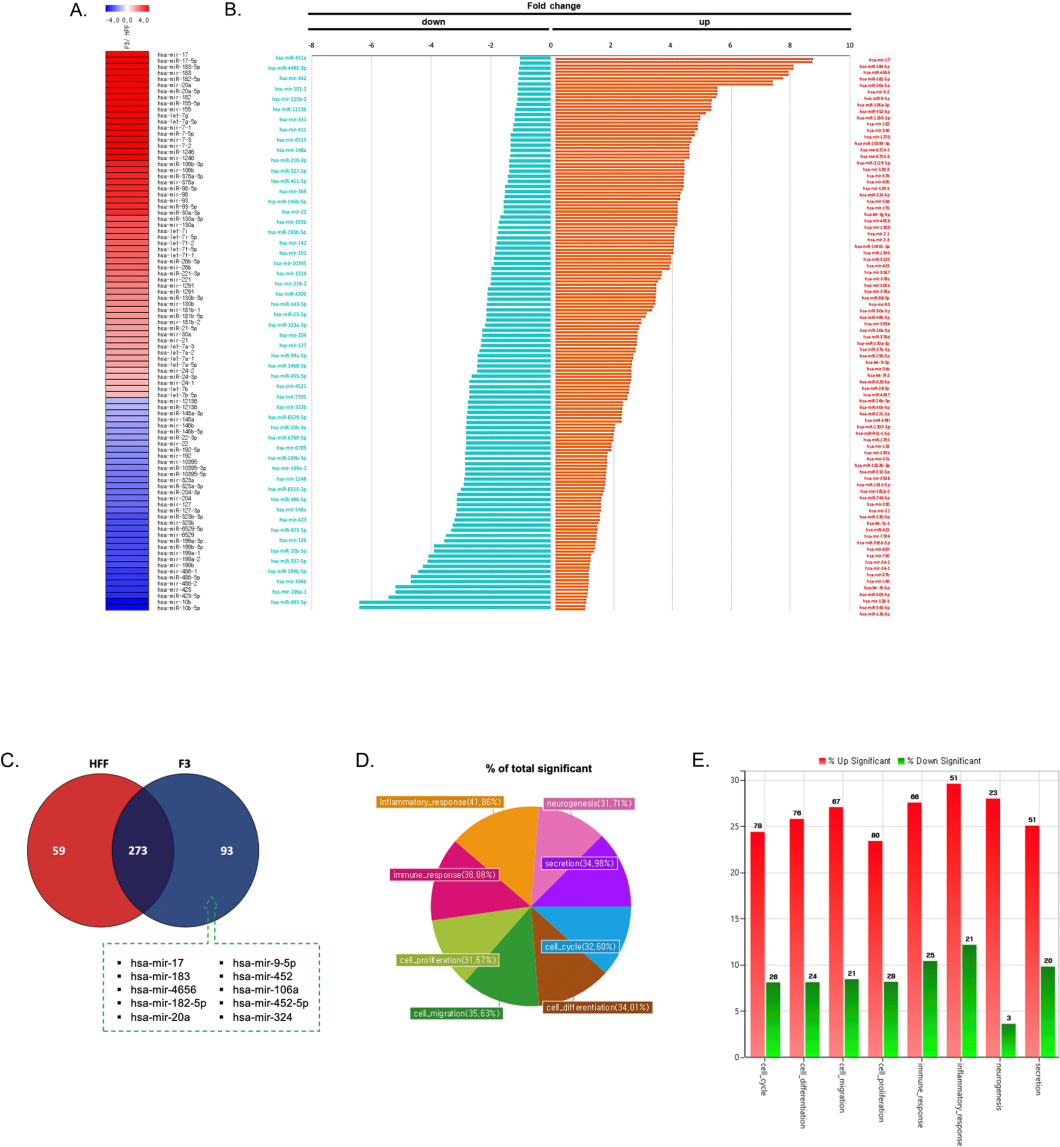

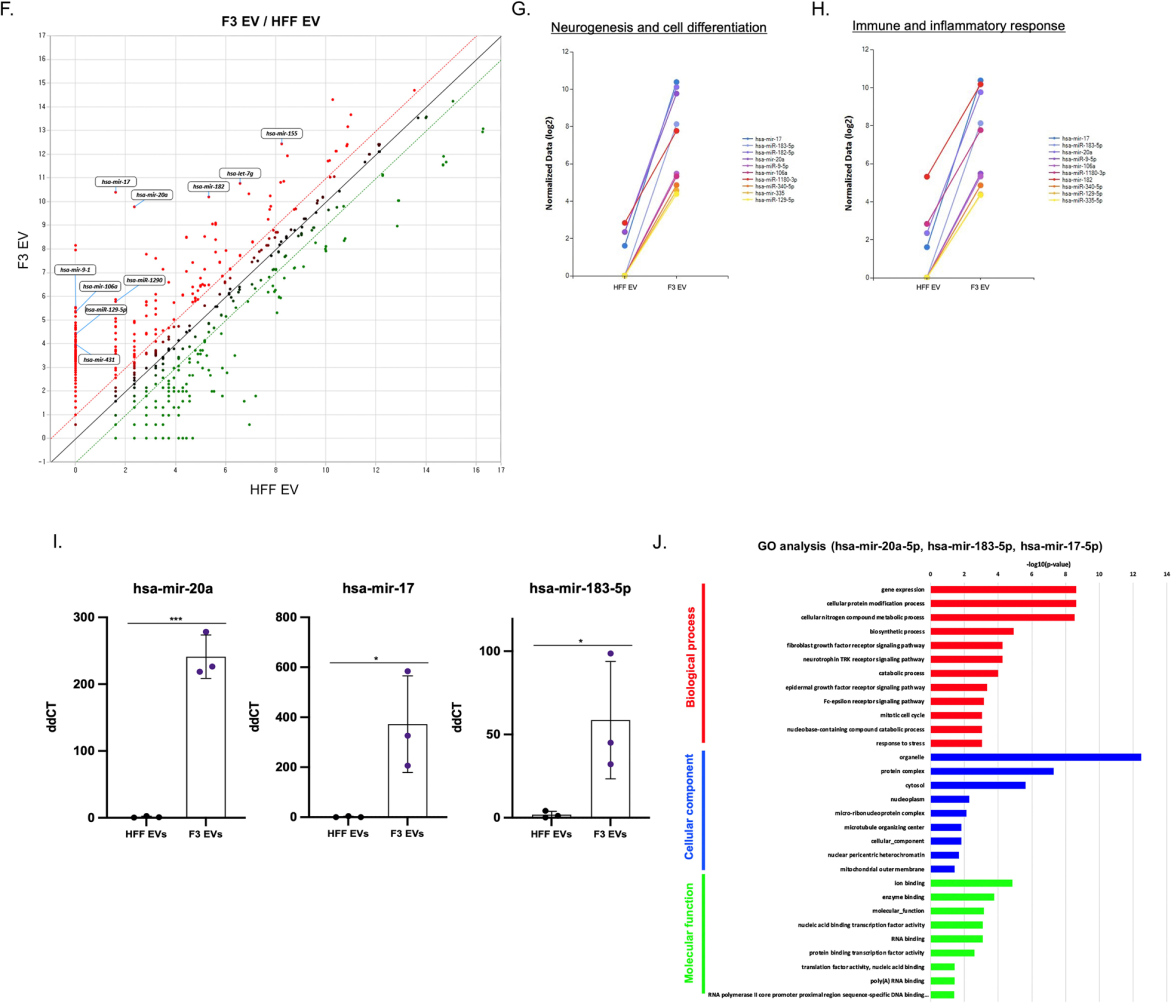
MiRNA expression profiles in F3- or HFF-derived EVs by small RNA sequencing. **A** Heatmap showing the differences in the miRNA expression profiles in F3- and HFF-derived EVs (total 92 miRNA). Red signal indicates high expression, and blue signal indicates low expression (normalized log2 values of 8 or more and two-fold change). **B** Summary diagram of miRNA differentially expressed in F3-derived EVs compared to HFF-derived EVs. **C** Venn diagram of the number of miRNAs expressed in EVs derived from F3 and HFF cells. The top 10 miRNAs uniquely expressed in F3-derived EVs compared to HFF-derived EVs were listed. **D** Pie chart of the percentages of involved miRNAs based on significantly changed gene categories. **E** Bar graph represents the number of miRNAs with significantly increased or decreased expression within the gene categories. **F** Scatter plot of the comparison of miRNA expression in EVs derived from F3 and HFF cells. Red dots indicate high expression, and green dots indicate low expression. **G**, **H** Expression plots of normalized log2 data were displayed in neurogenesis and cell differentiation categories, and in immune and inflammatory response categories. **I** Expression of selected miRNAs (hsa-mir-20a, hsa-mir-17, hsa-mir183-5p) significantly changed between F3- and HFF-derived EVs was validated using qPCR. (J) GO analysis was conducted for target genes (mRNAs) regulated by top three miRNAs (hsa-mir-20a-5p, hsa-mir-183-5p, hsa-mir-17-5p) that were differentially expressed between F3- and HFF-derived EVs. The GO terms were listed based on *p*-values (*p* < 0.05)

The quality of small RNAs were analyzed after isolation from EV samples (Additional file 1: Fig. S5). Then, the small RNAs were detected by next-generation sequencing to identify key small RNA cargoes of F3-derived EVs involved in their protective effect on PD. A total of 1434 miRNAs and 7851 long noncoding RNAs (lncRNAs) from F3- and HFF-derived EVs were analyzed (Additional file 2: Table S1 and S2). Among EVs secreted from F3 and HFF cells, differential miRNAs with normalized log2 values of 8 or more and two-fold change were displayed as a heatmap (Fig. 3A). Hsa-mir-17, hsa-mir-183, hsa-mir-20a, hsa-mir-182, and hsa-mir-155 were identified as the five most abundant miRNAs in EVs derived from F3 compared to HFF cells. Fold changes in EV miRNAs secreted from two different cell types were specifically identified by dividing them into increase or decreases in F3/HFF EV fold change factors. Hsa-mir-17, hsa-mir-183-5p, hsa-mir-4656, hsa-mir-182-5p, hsa-mir-20a-5p, and hsa-mir-9-2 were on top in the increased fold change values. In contrast, hsa-mir-451a, hsa-mir-4485-3p, hsa-mir-342, and hsa-mir-101-2 were decreased in the F3/HFF fold change (Fig. 3B). Further evaluation of the differential expression of miRNAs using Venn diagram showed that 273 miRNAs were expressed in both F3- and HFF-derived EVs. 93 miRNAs in F3-derived EVs and 59 miRNAs in HFF-derived EVs were found (Fig. 3C). The GO pie chart shows the percentage of genes with significantly altered expression among selected GO-related genes. The changes in the expression of the GO genes of interest were categorized as inflammatory response, neurogenesis, immune response, secretion, cell proliferation, cell cycle, cell migration, and cell differentiation (Fig. 3D). The percentages of significantly increased and decreased miRNAs compared to the control for each GO category are shown in Fig. 3E. Upregulated miRNAs based on F3/HFF fold-change values were approximately three-fold higher than downregulated miRNAs in each category. Differentially expressed miRNAs above the fold threshold line were identified using a scatter plot. Among the upregulated miRNAs in F3-derived EVs, miRNAs (hsa-mir-9-1, hsa-mir-106a, hsa-mir-129-5p, hsa-mir-431, hsa-mir-1290, hsa-mir-17, hsa-mir-20a, hsa-mir-182, hsa-let-7g, and hsa-mir-155) belong to the categories of cell differentiation, neurogenesis, and immune response (Fig. 3F). Especially, mir-9 and let-7 are known to involve the transition from induced pluripotent stem cells (iPSCs) to NSC [17, 21]. The top 10 miRNAs in the categories of neurogenesis and cell differentiation were compared between F3 and HFF cell-derived EVs with normalized data (Fig. 3G). The top 10 miRNAs in immune and inflammatory responses were then compared (Fig. 3H). Significant increases in miRNA expression levels were observed in F3-derived EVs in both categories. The most abundantly expressed miRNAs (hsa-mir-17, hsa-mir-20a, hsa-mir-183-5p) in F3-derived EVs compared to HFFs were verified by qRT-PCR analysis. In the case of HFF-derived EVs, there was almost no expression, whereas in F3-derived EVs, an expression difference of 200 to 300 times or more was confirmed (Fig. 3I). In order to identify possible pathways modulated by the top three differentially expressed miRNAs (hsa-mir-20a-5p, hsa-mir-183-5p, and hsa-mir-17-5p), GO enrichment analysis was performed for biological processes, cellular components, and molecular functions. To elucidate the protective effects of F3-derived EVs, significantly related biological processes were listed, particularly the fibroblast and epidermal growth factor receptor signaling pathways, the neurotrophin tropomyosin receptor kinase (TRK) receptor signaling pathway, and the response to stress that may be involved (Fig. 3J). Mir-182-5p and mir-183-5p are known to increase neurite outgrowth and protect dopaminergic neurons, mimicking glial cell line-derived neurotrophic factor effects [22].

For the lncRNA sequencing results, differential lncRNAs with normalized log2 values of 8 or more and a two-fold change were displayed as a heatmap (Additional file 1: Fig. S6A). The Venn diagram revealed that 1778 lncRNAs were expressed at both values using at least eight normalized log2 data. A total of 672 lncRNAs in F3-derived EVs and 441 lncRNAs in HFF-derived EVs were uniquely found (Additional file 1: Fig. S6B). The scatter plot shows the differentially expressed lncRNAs above the fold threshold line (Additional file 1: Fig. S6C). The GO categories of inflammatory response, neurogenesis, immune response, secretion, RNA splicing, DNA repair, cell cycle, cell migration, and cell differentiation were confirmed by the GO pie plot (Additional file 1: Fig. S6D). The percentages of IncRNAs from F3-derived EVs significantly increased and decreased compared to HFF-derived EVs for each GO category (Additional file 1: Fig. S6E). Significantly upregulated lncRNAs (APC, ROR2, NSUN2, EPHA6, SALL1, SEMA6A, CALML5, RANBP3L, PAXIP1, and SYT4) in the categories of neurogenesis, cell differentiation, and immune response were labeled. The top 10 lncRNAs in neurogenesis and cell differentiation categories were compared between F3- and HFF-derived EVs (Additional file 1: Fig. S6F). In addition, the top 10 lncRNAs in immune and inflammatory responses were compared (Additional file 1: Fig. S6G). A significant increase was observed in F3-derived EVs in both categories.

### Validation of EV tracing reporter activity and isolated EVs in F3-palm-tdTomato cells

**Fig. 4:**
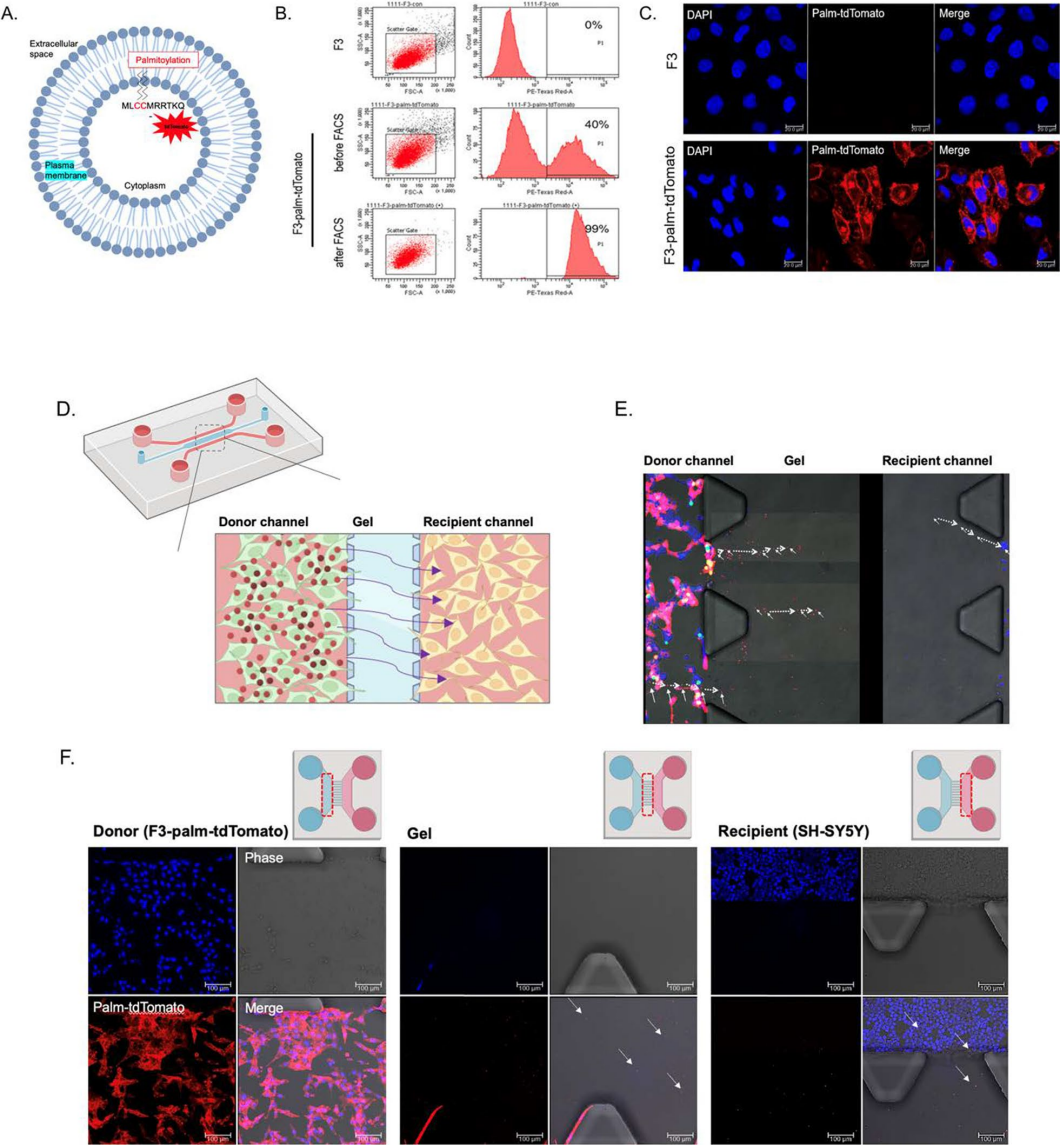
In vitro tracing of EVs secreted from palm-tdTomato virus-transduced F3 cell line in microfluidic device. **A** Schematic diagram of palm-tdTomato lentiviral vector for EV labeling. **B** TdTomato fluorescence positive F3 cells were sorted by flow cytometry after infection of palm-tdTomato lentivirus. The number of fluorescence positive cells were increased from 40 to 99%. **C** Confocal fluorescence images in cell-sorted tdTomato fluorescence positive F3 cells. Scale bar = 20 µm. **D** Schematic diagram of the two-channel microfluidic device. **E** F3-palm-tdTomato cells in donor channel and SH-SY5Y cells in recipient channel were co-cultured on a microfluidic device. Time-lapse imaging was used to track the migration of tdTomato fluorescence positive EVs in interchannel area (arrows) using time-lapse imaging. The imaging was performed for 6 times with 5 min intervals. **F** Each channel was monitored, showing tdTomato positive EVs (arrows) budding out from donor channel and migrating to the recipient channel using confocal microscopy. Scale bar = 100 µm

To track the behavior of EVs secreted from F3 cells, we labeled multiple EV populations with a palmitoylated-tdTomato (palm-tdTomato) lentivirus, tagged to the intracellular cell membrane area in the cytoplasm [23] (Fig. 4A). We adopted a lentivirus infection method to enhance intracellular delivery efficacy and long-term transgene expression of palm-tdTomato in F3 cells. Before sorting, tdTomato fluorescence-positive F3 cells showed 40% efficacy, and after sorting by FACS analysis, the cell population showed 99% of tdTomato-positive cells (Fig. 4B). Confocal microscopic images showed that tdTomato signals were consistently expressed in the F3-palm-tdTomato cells (Additional file 1: Fig. S7A), and the fluorescence intensity was measured for 7 days (Additional file 1: Fig. S7B). Fluorescence activities were stably maintained in F3-palm-tdTomato cells. We observed prominently bright signals in the cell membrane area in palm-tdTomato infected F3 cells (Fig. 4C).

Then, we isolated EVs from the supernatant of F3-palm-tdTomato cells. The EV sizes were uniformly distributed, with a peak diameter of approximately 120–200 nm (Additional file 1: Fig. S7C), and expressed intense tdTomato fluorescence controls (Additional file 1: Fig. S7D). Both exosomal protein markers, CD63 and CD81, were identified in F3- and F3-palm-tdTomato-derived EVs (Additional file 1: Fig. S7E). Then, the time conditions at which EVs were taken up into cells were evaluated using F3-palm-tdTomato cell derived EVs (Additional file 1: Fig. S7F). After 30 min, a sufficient amount of administrated EVs with tdTomato fluorescence signals was shown inside the SH-SY5Y cells.

Moreover, microfluidic devices were designed to trace EVs budding out from the donor cells and reaching the recipient cells (Fig. 4D). F3-palm-tdTomato cells in a donor channel and SH-SY5Y cells in a recipient channel were co-cultured. The EVs were traced using time-lapse live monitoring for 30 min. The EV transport expressed in tdTomato fluorescence signals from donor cells through the gel and to the recipient channel was captured (Fig. 4E). Magnified images of each channel also showed that the EV signal passed through the gel area to the recipient cells (Fig. 4F).

### In vivo tracing of implanted F3 cells or F3-derived EVs in mice using reporter activities

**Fig. 5:**
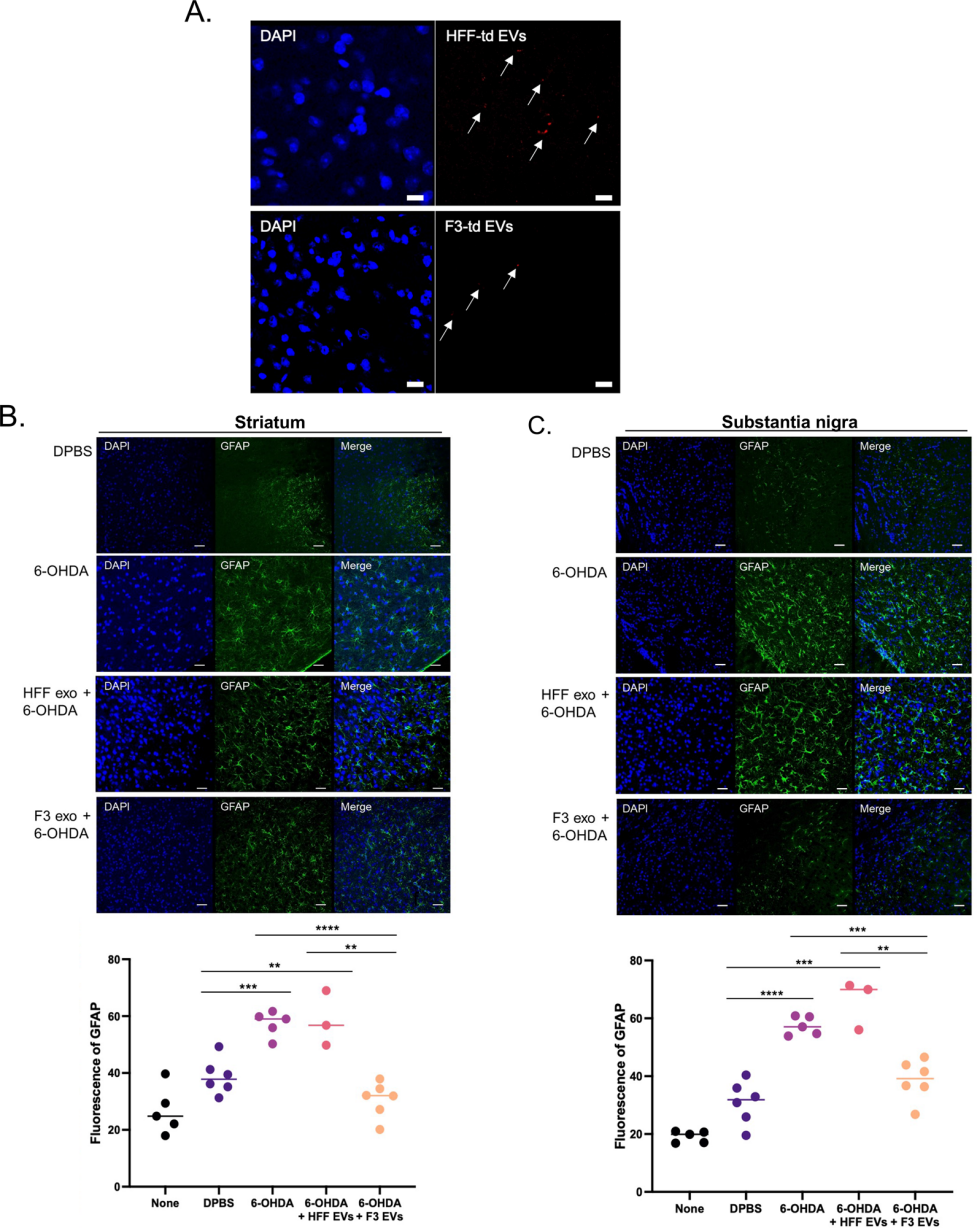

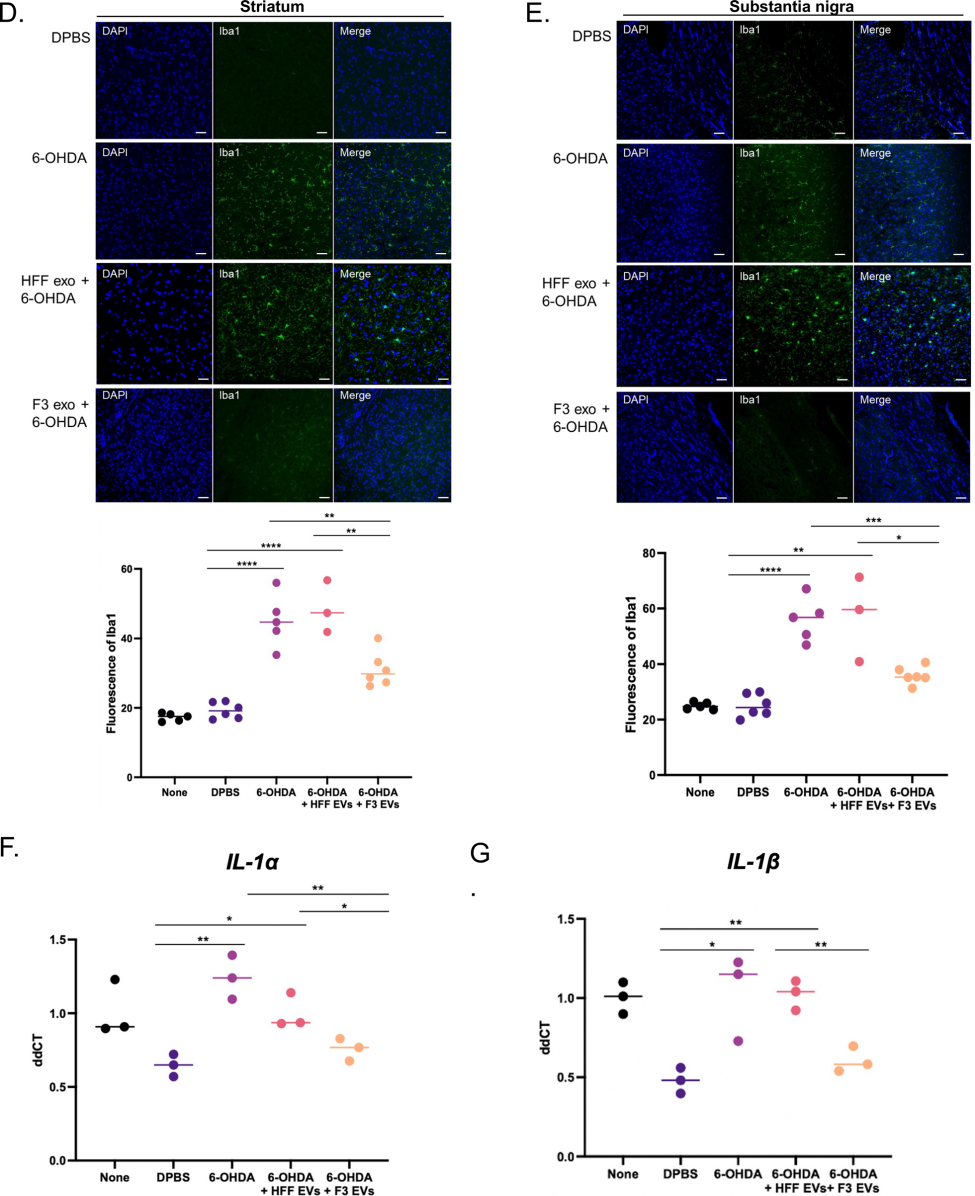
Anti-inflammatory effect of F3-derived EVs in 6-OHDA induced PD mouse model. 6-OHDA (3 mg/mL) was intracerebrally injected with F3- or HFF-derived EVs into the left side of the SN region of the brain. **A** F3- and HFF-palm-tdTomato-derived EVs (arrows) were observed 24 h after injection of EVs into the SN region of the brain in PD. **B**, **C** Reactive astrocytes were identified by GFAP staining in the SN and striatum. The total area of GFAP-positive fluorescence was quantified. **D**, **E** Reactive microglia were observed by Iba1 staining in the SN and striatum in a PD mouse model. The total area of the Iba1-positive fluorescence was quantified. **F**, **G** RNA expression levels of *IL-1α* and *IL-1β* in SN tissues were obtained by qPCR analysis. 6-OHDA-induced reactive glial cells and intracellular inflammatory cytokine levels were reduced by F3-derived EV treatment Scale bar = 50 µm

To determine the location of F3-or F3-derived EVs, luciferase-expressing F3 cells or palm-tdTomato-expressing F3 cells were intracerebrally injected into the SN region of the mouse brain. First, to verify the viability of F3 cells transplanted into mice in vivo, F3 cells were infected with the elongation factor 1 alpha promoter-driven luciferase 2-green fluorescent protein (EF1a-luc2-GFP) virus (Additional file 1: Fig. S8A, B). The luciferase activity of implanted F3 cells was identified in live mice and isolated brains on days 1, 3, and 6 using the IVIS imaging system (Additional file 1: Fig. S8C). The injected cells were located at SN regions and survived for a week. Also, palm-tdTomato-expressing F3 cells were implanted into the SN area of the normal brain. The EV signals were observed from the image at the injection site and even in the region adjacent to the injection site (Additional file 1: Fig. S8D). EVs were identified by vesicle-shaped tdTomato signals in the membrane region of the implanted F3 cells using super-resolution microscopy (Additional file 1: Fig. S8E). Through the CLARITY [24], injected F3-palm-tdTomato cells were well identified not only in a single thin section, but also in the 1 mm thick region containing the SN (Additional file 1: Fig. S8F). In addition, EVs isolated from HFF- and F3-palm-tdTomato cells were directly injected into the SN region of the brain, and EVs derived from F3 and HFF cells were verified by tdTomato fluorescence signal (Fig. 5A).

### Anti-inflammatory effect of F3-derived EVs on 6-OHDA induced PD mouse model

To establish PD in mice, 6-OHDA was intracerebrally injected into the left side of the brain in the SN region. The apomorphine-induced behavior analysis confirmed the neurotoxic effect of 6-OHDA. PBS-injected mice turned without any restriction in direction while 6-OHDA injected PD models exhibited a rotational pattern in only one direction (Additional file 1: Fig. S9A).

Reactivity of astrocytes and microglia and changes in pro-inflammatory cytokines were identified. The reactive astrocytes were increased in the striatum and SN area of the brain 1 week after 6-OHDA injection. In contrast, the reactivity was significantly attenuated in the F3-derived EV-treated group compared to the HFF-derived EV-treated group in the striatum (Fig. 5B) and SN regions (Fig. 5C). In addition, microglia were activated in the striatum and SN of PD models, showing an increase in the Iba-1 marker. Reactivity was upregulated in the 6-OHDA only or HFF-derived EV-treated groups, but the F3-derived EV-treated group showed no increase in Iba-1 induced by 6-OHDA in the striatum (Fig. 5D) and the SN regions (Fig. 5E). When the expression of pro-inflammatory cytokines around the SN region was measured in PD mice, 6-OHDA induced an increase in *IL-1α* and *IL-1β.* Interestingly, F3-derived EVs treated with 6-OHDA showed that the levels of both cytokines were significantly downregulated, compared to the HFF-derived EV-treated group (Fig. 5F and G). It was confirmed that the EVs secreted from F3 cells have a protective action against neuroinflammation.

### Protective effect of F3-derived EVs on dopaminergic cell viability in 6-OHDA induced PD mouse model

**Fig. 6:**
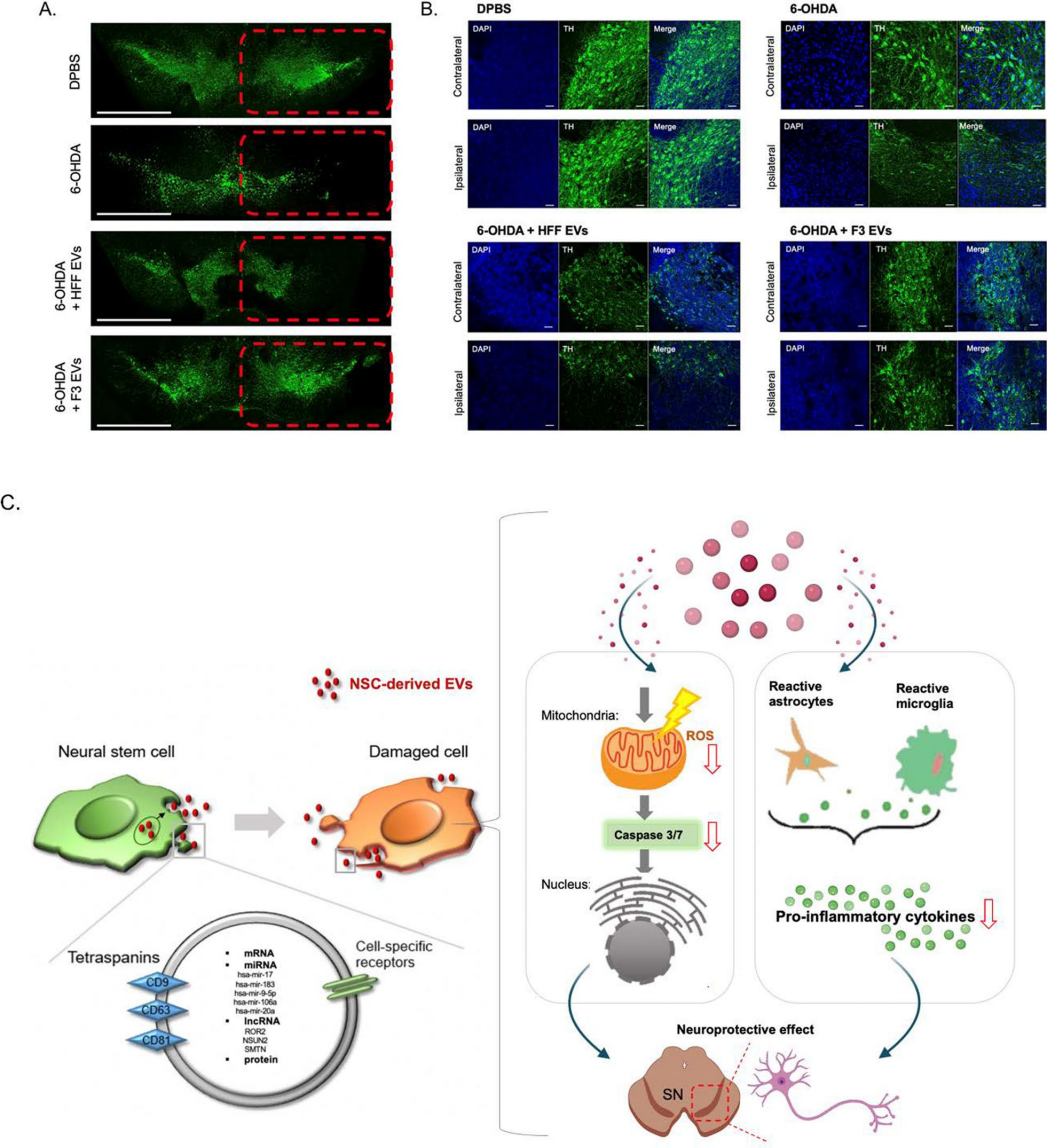
Protective effect of F3-derived EVs on dopaminergic cell viability in 6-OHDA- induced PD mouse model. **A** Immunofluorescence staining of TH was performed to detect viability of TH-positive dopaminergic neurons in SN. The red box indicates ipsilateral regions. scale bar = 1 mm. **B** Magnified images of the ipsilateral and contralateral sides of the SN were shown. Degeneration of TH-positive dopaminergic neurons by 6-OHDA was attenuated by F3-derived EVs treatment in SN. N = 3, scale bar = 50 µm. **C** Schematic diagram for the protective effect of F3-derived EVs against 6-OHDA was shown. Anti-oxidative stress and anti-inflammatory effects of EVs containing miRNAs that are involved in dopaminergic neuronal protection were displayed. *NSC* neural stem cell. *SN* substantia nigra, *EV* extracellular vesicles, *ROS* reactive oxygen species

To determine the protective effect of the degenerating dopaminergic neurons by F3-derived EVs, we measured the viability of dopaminergic neurons. In 6-OHDA-induced PD mice, a reduction in TH-positive neurons in the ipsilateral SN was observed (Fig. 6A). A decrease in the TH-positive signal was also observed in the ipsilateral striatum (Additional file 1: Fig. S9B). F3-derived EVs treated with 6-OHDA dramatically prevented degeneration of dopaminergic neurons in the SN, compared to the HFF-derived EVs treated group after a week of injection (Fig. 6A, B). Together, our results demonstrated the protective effect of F3-derived EVs by anti-oxidative stress and anti-inflammatory effects in 6-OHDA induced PD pathology (Fig. 6C).

## Discussion

The results of the present study indicate that NSC-derived EVs attenuate the ROS-induced apoptotic pathway and neuroinflammation, the major pathological features of PD. In the condition of 6-OHDA-induced dopaminergic neurons, cell death mainly occurred by the generation of ROS. F3-derived EVs were sufficient to suppress neuronal damage caused by 6-OHDA treatment. F3-derived EVs also protected the survival of degenerating dopaminergic neurons in 6-OHDA-treated animal models of PD. We speculated that the cell survival- or neurogenesis-associated miRNAs or lncRNAs may be involved in the neuroprotective effect of PD.

NSC grafts serve as a natural source of potent biological agents that can promote functional restoration in the central nervous system (CNS) tissue following acute or chronic tissue injury. NSCs derived from fetal, neonatal, and postnatal tissues are pluripotent and can differentiate into the main types of CNS cells, including neurons, astrocytes, and oligodendrocytes [14]. One of the ways in which NSCs contribute to regeneration in damaged brain tissues involves paracrine function by secreting neurotrophic or growth factors. EVs act as individual message packets containing RNAs and proteins and migrate to proximal and distant recipient cells to alter their function and phenotype [25]. NSC-derived EVs with stem cell properties could potentially be used as an alternative cell-free therapy for the treatment of PD. In particular, EVs have the substantial advantages of being easier to produce and store than cells, and avoid the regulatory issues faced by NSCs [16].

Recent studies showed the therapeutic effect of exosomes derived from mesenchymal stem cells (MSCs) to the recovery of PD. MSC-derived exosomes promoted the angiogenesis and alleviate the damage of human brain microvascular endothelial cells. The symptoms of PD improved as a functionally integrated network between neurons and vascular cells that regulate neuronal survival and vascular homeostasis was restored [26]. Moreover, NSCs offer advantages over MSCs, as they are the most potent cells capable of differentiating into the neural lineage. According to a recent study, NSC-derived EVs showed more effective neuroprotective roles and improved functional outcomes in stroke animal models compared to MSC-derived EVs [27]. Therefore, NSC-derived EVs show high potential for developing PD therapeutics compared to cell transplantation and other cell types.

In this study, the increased viability of SH-SY5Y cells after we co-cultured F3 cells revealed that indirect factors act as neuroprotectors (Additional file 1: Fig. S1B). We then hypothesized the beneficial effect of direct treatment of EVs, a well-known communicator between cells.

Here, we verified that F3-derived EVs were effectively taken up into SH-SY5Y cells using palm-tdTomato signals. It is an ideal EV labeling method that shows sufficient signals to detect nanoscale EVs, and to reduce potential disturbance to EV surface protein composition. The proper half-life of fluorescence signals is also consistent with that of EVs. The most commonly used dyes to label EVs, such as lipophilic dyes, have a much longer half-life than EVs, potentially leading to false-positive signals [23]. We observed small spherical signals around the cell membranes that were predicted to be EVs (Additional file 1: Fig. S8D). The EV-tracing microfluidic device revealed that the cells indirectly communicated with each other through the extracellular matrix gel [28] (Fig. 4D–F).

We further investigated the neuroprotective role of F3-derived EVs and possible signaling mechanisms against 6-OHDA-induced cellular damage. The generation of intracellular ROS by 6-OHDA is an initial event that finally leads to neuron degeneration [29]. Therefore, inhibiting ROS production is an effective strategy for reducing oxidative stress-mediated cell death. F3-derived EVs significantly alleviated the ROS-mediated apoptotic pathway (Fig. 1). In PD models, reduced TH positive dopaminergic neurons are one of the pathological factors that reduce DA synthesis [30]. While only F3-derived EVs maintained the viability of dopaminergic neurons (Fig. 6), the data suggest that bioactive components contained in EVs derived from NSCs, rather than fibroblasts may play an important role in protecting against 6-OHDA-induced damage.

Moreover, reactive glial cells with elevated pro-inflammatory mediators in the SN of PD patients were detected by a postmortem study [11]. Increased inflammasomes have also been shown in PD animal models and in the plasma of patients [31]. In particular, it was reported that the imbalance between Th17 cells and regulatory T cells acts as a pro-inflammatory factor, triggering cytokines and inducing glial hyperactivation [32]. The increased interaction of microglia and peripheral immune cells can induce the overproduction of pro-inflammatory cytokines associated with the neuroinflammatory cascade and neurodegenerative diseases. The reduced pro-inflammatory cytokines and chemokines with F3-derived EV treatment (Fig. 2 and Additional file 1: Fig. S4) were inferred as a result of decreased microglial activity. Also, the inflammatory cytokines (TNF-a, IL-1b, IL-6, IL-12, IL-1) and chemokine (CCL2, CXCL10), which are known as downstream factors of nuclear factor-kappa B (NF-κB), were attenuated after the administration of F3-derived EVs compared to LPS group [33]. Through these results, it can be considered that the NF-κB pathway effectively reduced through F3-derived EVs as a mechanism of astrocyte reactivity induced by 6-OHDA. In the PD model, the reactivity of 6-OHDA-induced glial cells in the SN was significantly upregulated, but F3-derived EVs reduced the reactivity of microglia and astrocytes (Fig. 5). NSC-derived EVs could control neuroinflammation by decreasing glial cell reactivity and pro-inflammatory cytokine release in a PD model.

It has been reported that the RNA cargo of EVs can alter gene expression and function of recipient cells [34]. In particular, the beneficial effects of specific miRNAs on neurons have been investigated. Representative miRNAs that play essential roles in neurogenesis are let-7, miRNA-124, and miRNA-9, which are known to promote the differentiation of NSCs into neuronal cells [21, 35, 36]. In addition, miRNA-188-3p-enriched exosome treatment has been suggested as a new therapeutic target for PD by suppressing autophagy and pyroptosis [37].

In this study, differentially expressed small RNAs in F3-derived EVs compared to HFF-derived EVs were identified, and the characteristics of NSC-derived EVs had a protective effect in the PD model (Fig. 3 and Additional file 1: Fig. S6). The miRNAs mentioned above, especially miRNA-9 and let-7, were highly expressed in F3- compared to HFF-derived EVs (Fig. 3A and B). Interestingly, hsa-mir-17, hsa-mir-183-5p, and hsa-mir-20a were identified to be involved in both neurogenesis and cell differentiation (Fig. 3G) and immune response categories (Fig. 3H). The GO analysis of target genes by the top three miRNAs revealed a significant relationship with the neurotrophin TRK receptor signaling pathway, fibroblast and epidermal growth factor receptor signaling pathway, and response to stress (Fig. 3J). The effects of neurotrophins in PD are essential for dopaminergic neuron survival, but neurotrophic activities are blocked by alpha-synuclein in pathologic conditions [38]. Alternations in the activation of TRKB correlate with relevant pathways such as neuronal differentiation and synaptic plasticity [39]. Interestingly, hsa-mir-183-5p and hsa-mir-182-5p mimic the effect of glial cell line-derived neurotrophic factor, which potently increases dopaminergic neuron survival in PD. These miRNAs function by decreasing the expression of FOXO1 and FOXO3. Inhibition of FOXO1 and FOXO3 transcription factors plays a role in neurite regeneration and the prevention of oxidative stress-induced apoptosis [22]. The results indicated that the protective effect against 6-OHDA toxicity by the miRNA cargo of EVs regulates inflammation, oxidative stress, and neuronal regeneration.

## Conclusions

Overall, NSC-derived EVs transmit the functional biomolecules of NSCs to damaged target cells, and as a result, contribute to protecting degenerating neurons through antioxidant, anti-apoptosis, and inflammation inhibition (Fig. 6C). Therefore, NSC-derived EVs offer a potential practical clinical option for PD prevention.

## Methods

### Cell culture

SH-SY5Y cells, a human dopaminergic neuroblastoma cell line, and human foreskin fibroblasts (HFF) were purchased from the American Type Culture Collection (ATCC). F3 cells, a human fetal telencephalon (15 weeks gestation)-derived immortal NSC line, were provided by Prof. Seung U Kim from Chung-Ang university of Korea. BV2 cells, a type of microglial cell derived from C57/BL6 murine, were purchased from the AcceGen. The cells were cultured in Dulbecco’s modified Eagle’s medium (DMEM) supplemented with 10% fetal bovine serum (FBS) and 1% penicillin/streptomycin in a 5% CO_2_ atmosphere at 37 °C.

### Preparation of EVs

EV-depleted FBS was collected by ultracentrifugation at 117,092*g* for 16 h at 4 °C. F3-palm-tdTomato stable cells were incubated in DMEM containing 10% EV-depleted FBS for 48 h. The supernatant of cells was collected, and the remaining cells and debris were removed by centrifugation at 2000*g* for 30 min. The supernatant (0.5 L) was collected and concentrated using a sterile membrane, T-series cassette (Pall Life Sciences) as the tangential flow filtration (TFF) system. The filters were washed three times with DPBS to adjust the pH to 7.4. The supernatant was then placed on the membrane. Particle sizes below 15 nm were filtered out of the system. 0.5 L of media containing EVs was concentrated to approximately 15 mL. Then, the final EV was isolated using a total EV Isolation kit (Invitrogen) and then resuspended in 1× PBS. The amount of protein was estimated using a bicinchoninic acid assay (BCA; Thermo Scientific).

### Western blot analysis

The concentration of isolated EVs was measured using a bicinchoninic acid assay (Thermo Scientific). Equal amounts of protein were resolved by SDS-PAGE and transferred onto polyvinylidene fluoride membranes (Millipore). The membrane was probed with primary antibodies against anti-rabbit CD63 (1:1000 dilution; Santa Cruz Biotechnology), anti-mouse CD81 (1:1000 dilution; Santa Cruz Biotechnology), anti-rabbit Calnexin (1:1000 dilution; abcam), anti-rabbit HSP70 (1:1000 dilution; abcam), anti-rabbit CD9 (1:1000 dilution; abcam), and anti-TSG101 (1:1000 dilution; abcam) overnight at 4 °C. Membranes were incubated with HRP-conjugated anti-rabbit and anti-mouse secondary antibodies (1:10,000 dilution; Santa Cruz Biotechnology) for 2 h at 25–27 °C, and proteins were visualized using a chemiluminescence detection system (Promega).

### Nanoparticle tracking analysis

For quantification, an LM10 nanoparticle tracking analysis (NTA) system (Nanosight) was used to measure the EV size distribution and particle number. The 10 μg of EVs were diluted in PBS and analyzed.

### Assessment of live and dead cell populations

Cytotoxic effects of 6-OHDA on SH-SY5Y cells were detected using a Live/Dead Viability/Cytotoxicity kit (ThermoFisher). This assay kit provides simultaneous determination of live and dead cells based on a two-color fluorescence detection method. Live cells labeled with calcein AM were detected with green fluorescence (530 nm) and dead cells labeled with ethidium homodimer (EthD-1) were stained with red fluorescence (585 nm). The stained cells were washed with Dulbecco’s phosphate-buffered saline (DPBS; Gibco). The same number of cells was then removed from the plates. The fluorescence intensities of red and green were measured using a fluorescence microplate reader (Glomax).

### Cell viability assay

SH-SY5Y cells (1 × 10^5^ cells) were seeded in 96-well plates and incubated overnight. After treatment with F3-derived EVs for 30 min, the cells were incubated with 6-OHDA (Sigma Aldrich) in 100 μL of media. Cell viability was measured using the cell counting kit-8 (CCK-8) assay system (DOJINDO). Absorbance was measured at 450 nm.

### Measurement of intracellular ROS by fluorescence microplate reader

SH-SY5Y cells (1 × 10^5^ cells) were treated with F3-derived EVs for 30 min and then incubated with 6-OHDA for 24 h in a 96-well plate. The cells were then collected and incubated with 20 µM 2,7-dichlorofluorescein diacetate (DCF-DA, Sigma-Aldrich, St. Louis MOUSA) for 1 h at 37 °C in the dark. Intracellular ROS levels were quantified using a fluorescent microplate reader (485 nm excitation and 535 nm emission, Varioskan Flash, Thermo Scientific).

### Measurement of mitochondrial membrane potential (MMP)

MMP was determined using the JC-1 mitochondrial membrane potential assay kit (Abcam). SH-SY5Y cells (1 × 10^5^ cells) were treated with JC-1 (10 µmol/L) for 1 h at 37 °C. The fluorescence intensities of red and green were measured with a fluorescence microplate reader (Glomax) upon emission wavelengths for detecting red (590 nm) and green (530 nm) signals.

### Measurement of caspase-3/7 activity

Caspase-3/7 activity was measured using Caspase-Glo 3/7 Assay Systems (Promega) in the SH-SY5Y cell line (1 × 10^5^ cells). EV-treated cells were incubated with 6-OHDA for 24 h. Caspase-Glo 3/7 reagent was added directly to the cells in 96-well plates and incubated for 30 min at 25–27 °C in the dark according to the manufacturer’s instructions. Luciferase activity was measured using Glomax (Promega).

### Flow cytometric analysis using propidium iodide (PI)

Cytotoxicity was determined using the PI fluorescence method. SH-SY5Y cells (1 × 10^6^ cells) were seeded on 6-well plates and treated with F3-derived EVs for 30 min on the following day. Following EV treatment, the cells were incubated with 6-OHDA for 24 h. Then, 1 × 10^6^ detached cells were labeled with PI (Sigma Aldrich) for 10 min at 25–27 °C according to the manufacturer’s instructions. After 10 min, the cells were analyzed for cytotoxicity using the FACSCalibur flow cytometer (BD bioscience).

### LPS stimulation

The BV2 cells (5 × 10^5^ cells) were seeded on 6 well plate. Next day, the different concentration of LPS (0.01, 0.05, 0.1, 0.5, 1, 5 μg/mL) was treated on BV2 cells for 24 h. The control group was treated the same amount of DPBS in the same condition.

### Transwell assay

Transwell inserts with a PET membrane of 0.4-µm pore size (BD Bioscience) were used. The pre-warmed culture medium was placed in each well and inserted for pH equilibrium. SH-SY5Y cells (1 × 10^5^ cells) were seeded on the inserts and BV2 cells (2 × 10^5^ cells) were seeded on 12 well plates. Then, the co-culture and treatment of EVs and 6-OHDA were processed according to the schedule shown in Fig. 2C.

### Fabrication of microfluidic device

A microfluidic device was fabricated by bonding microchannel-patterned poly-dimethysiloxane (PDMS; Sylgard 184; Dow Corning) to a glass coverslip. The photoresist SU-8 (MicroChem) was used as a master mold, fabricated by a conventional soft-lithography process, to replicate the microchannel-patterned PDMS. The PDMS elastomer was thoroughly mixed with the curing agent at a 10:1 ratio. Then, the mixture was poured onto the wafer and incubated in an oven at 80 °C for 1.5 h. After curing, the PDMS replica was removed from the wafer, and all reservoir patterns on the PDMS replica were punched using dermal biopsy punches. The sterilized PDMS replica and glass coverslip were bonded together via oxygen plasma (Femto Science) and placed at 80 °C in an oven for at least 24 h to restore the hydrophobicity of the microchannel surfaces.

### Microfluidic cell culture assay

Type 1 collagen ECM (2 mg/mL; BD Biosciences) was gently injected into the hydrogel channel and gelled for 30 min in a humid chamber at 37 °C. Medium was then added into the channels to prepare cell seeding. F3-palm-tdTomato cells were seeded in one reservoir of the channel (left) and SH-SY5Y cells were in the other channel (right). After cell attachment, conditioned medium was added in both channels. After 24 h, the microfluidic devices were imaged with confocal microscopy for real-time imaging.

### RNA isolation and RT-PCR

Total RNA from cells and the substantia nigra of the mouse brain was extracted with TRIzol reagent according to the manufacturer’s instructions (Ambion). The cDNA samples were amplified using primers based on the sequences listed below. Then, agarose gel electrophoresis was performed to analyze the PCR products. Primer information: *IL-1α* forward-5′ CGCTTGAGTCGGCAAACAAA 3′ reverse-5′ TGATACTGTCACCCGGCTCT 3′; *IL-1β* forward-5′ CTCCATGAGCTTTGTACAAGG 3′ reverse-5′ TGCTGATGTACCAGTTGGGG 3′; *β-actin* forward 5′ AAGACCTCTATGCCAACACAGT 3′, reverse 5′ GCTCAGTAACAGTCCGCCTA 3′.

### Quantitative PCR (qPCR)

cDNA samples were diluted and mixed with SYBR green master mix (Takara) before loading as technical triplicates for qPCR on an Applied Biosystems 7500 system. Primers are specified above (in Section “RNA isolation and RT-PCR”).

### Cytokine release assay by ELISA

The cells were seeded in plates and treated with lipopolysaccharide (LPS) for 24 h. After saline, HFF-derived EVs, or F3-derived EVs were treated, cultured media were collected, and secretion of TNF-α was measured using an ELISA kit following the manufacturer’s instructions (Thermo Scientific). The absorbance at 450 nm was measured using a microplate reader (Glomax). Results are expressed as pg/mL of cytokine.

### Lentivirus preparation and infection

The 293FT cells (ATCC), derived from 293F cells and modified to stably express the SV40 large T-antigen, were plated in a 100-mm^2^ dish in DMEM supplemented with 10% FBS. 293FT cells were transfected with the Palm-td lentiviral vector and lentivirus packaging vectors (REV, PMDLg, and Env) using Lipofectamine 2000 and diluted in OPTI-MEM medium (Gibco). Transfected cells were incubated in DMEM supplemented with 10% FBS for 48 h. After supernatant collection, cells and debris were removed by centrifugation at 2000×*g* for 30 min. Purified virus stocks were titrated using the Lenti-X qRT-PCR Titration Kit (Clontech). F3 cells were plated in a 100-mm^2^ dish with 800 μL of fabricated Palm-td lentivirus and 8.8 μL of polybrene (10 mg/mL, Santa Cruz Biotechnology), and were treated with DMEM containing 10% FBS. Completed F3-palm-tdTomato cells were washed with fluorescence-activated cell sorting (FACS) buffer (phosphate-buffered saline [PBS] solution containing 5% fetal bovine serum [FBS]). After centrifugation, the collected cells were resuspended in the FACS buffer. The purity of tdTomato fluorescence-positive cells was quantified by FACS (BD Immunocytometry System, CA) analysis.

### Assessment of fluorescence intensity stability

F3-palm-tdTomoato cells were plated in 24-well plates and 96-well plates and incubated for different lengths of time (days 1, 3, and 7). Confocal microscopy (Leica Microsystems) and a fluorescence microplate reader (Varioskan flash, Thermo Scientific) were used to detect fluorescence intensity and stability. Unlabeled F3 cells were used as controls.

### Mice

Male C57BL/6 mice (6-weeks old) were housed at constant temperature (25–27 °C) and humidity-controlled animal cages. All animal experiments were approved by the Institutional Animal Care and Use Committee of Seoul National University (IACUC; SNU-160923-4-7).

### Cytokine array

After EV treatment and LPS stimulation, BV2 cells for each group were washed with DPBS and collected. Proteins were isolated using RIPA buffer (thermo) according to the manufacturer’s protocol. The concentrations were measured using BCA assay kit (thermo). The 200 µg of each sample were used, and the cytokine array was proceeded using proteome profiler mouse cytokine array kit (R&D systems) according to the manufacturer’s protocol. Captured proteins on the array membrane were visualized using chemiluminescent detection reagents and chemiluminescent imaging system (GE, Amersham 680).

### Intracerebral injection

Male C57BL/6 mice (6-weeks old) were housed at constant temperature (25–27 °C) and humidity-controlled animal cages. All animal experiments were approved by the Institutional Animal Care and Use Committee of Seoul National University (IACUC). For surgery, the mice were anesthetized with isoflurane inhalation and placed on a stereotactic apparatus. A hole was drilled in the skull with needles at specific lateral coordinates (anterioposterior (AP) = −  3.2 mm; mediolateral (ML) = 1.5 mm; Dorsoventral (DV) = −  4.2 mm). Three microliters of F3-palm-tdTomato cell-derived EVs (100 mg/mL) were stereotaxically injected into the SN using a 10 µL Hamilton syringe. The needle was then left in place for another 5 min to allow complete diffusion before being slowly retracted. After surgery, mice were recovered near a heat lamp. Cyclosporin A (5 mg/kg) was administered intraperitoneally every day after transplantation to suppress the immune response against the human F3 cell line derived EVs.

### 6-OHDA induced PD mouse model

6-OHDA was prepared in saline and kept on ice during surgery. Mice were anesthetized with isoflurane and placed in a stereotaxic frame for mice. To achieve a unilateral lesion of the nigrostriatal pathway, mice received 2 µL of 6-OHDA stereotaxically injected into the SN. All 6-OHDA lesions were performed unilaterally (left) with the contralateral (right) hemisphere serving as a histological control. The following co-ordinates (relative to bregma) were used: anterioposterior (AP) = − 3.2 mm; mediolateral (ML) = 1.5 mm; Dorsoventral (DV) = − 4.2 mm with a flat skull position.

### Apomorphine induced behavior analysis

Before the behavioral test, mice were habituated to the testing room for at least 1 h. rotational activity induced by apomorphine is commonly used to evaluate the functional consequence of dopamine neuronal loss induced by 6-OHDA. After intraperitoneal injection of apomorphine hydrochloride (0.05 mg/kg, Sigma), they were placed in the transparent cylinders to acclimate for 5 min before the test. The circling response to apomorphine was evaluated by counting the number of apomorphine-induced contralateral rotations in 30 min.

### IVIS spectrum in vivo imaging system

To visualize the transplanted cells, F3 cells were infected with lentivirus to express the luciferase gene using the method described in the section “Lentivirus preparation and infection”. For the acquisition of bioluminescence images, mice were anesthetized with isoflurane inhalation. d-Luciferin (Promega) was diluted to 3 mg/100 µL in saline and 0.6 mg of d-Luciferin was intravenously administered. An IVIS-100 imaging system (Perkin Elmer) was used, and the images were acquired by integrating light for 30 s. The brains were then isolated from mice and imaged using the IVIS-100 imaging system.

### Brain tissue sampling

Brains were collected and microdissected coronally into the half. One part of the brain containing the substantia nigra region was placed in 4% paraformaldehyde (PFA) 8 for 48 h, and then made into paraffin block for immunofluorescence imaging. The other part of the brain was placed in Trizol for RNA isolation.

### Immunofluorescence imaging for tissue sections

Paraffin-embedded brain tissues were sectioned at 4-μm thickness. Deparaffinization was achieved using xylene and decreasing concentrations of ethanol. Tissue sections were subjected to antigen retrieval using citrate buffer (pH 6.0) at boiling temperature (above 100 °C) for 10 min. Following rinsing with Tris-buffered saline (TBS), the sections were incubated in blocking buffer containing TBS with 0.5% bovine serum albumin for 1 h at 25–27 °C. Slides were then incubated with the primary antibody in blocking buffer overnight at 4 °C. The next day, the slides were washed with TBS and stained with Alexa Fluor secondary antibodies (Thermo Fisher Scientific). Sections were rinsed again and stained with DAPI (1:100; Invitrogen) before being cover-slipped with mounting medium. The primary antibodies used were rabbit Iba1 (1:200; Abcam), mouse glial fibrillary acidic protein (GFAP; 1:200; Cell Signaling Technology), and rabbit TH (1:100; Abcam).

### CLARITY technique for brain tissue clearing

Mice were anesthetized with isoflurane inhalation and perfused intracardially with cold DPBS and fixed with 4% paraformaldehyde for 48 h. The brains were transferred into a hydrogel monomer solution with 4% acrylamide and 0.25% photoinitiator 2,20-Azobis[2-(2-imidazolin-2-yl)propane] dihydrochloride; Wako Chemicals) for 24 h at 4 °C. Hydrogel-infused brains were degassed using nitrogen in a vacuum desiccator for 3 min and incubated in a 37 °C water bath for 2 h. Excess hydrogel was removed from the polymerized 1 mm sections of whole brains. The brain sections were moved into a clearing solution (4% SDS in 200 mM boric acid in H_2_O, pH 8.5) in an electrophoresis chamber (Logos Biosystems), and 45 V was applied at 37 °C. The cleared brains were washed with DPBS overnight. After several washing steps, the samples were immersed in a CLARITY mounting solution (Logos Biosystems).

### Total RNA isolation from F3- and HFF-derived EVs for sequencing

The total exosomal RNA extraction was performed using Maxwell RSC miRNA from tissue and plasma or serum kit (Promega) according to the manufacturer’s protocols. Total RNA concentration was calculated using Quant-IT RiboGreen (Invitrogen). To determine the DV200 (% of RNA fragments > 200 bp) values, samples were run on the *TapeStation RNA screentape (Agilent).*

### Exosome small RNA sequencing

Total RNA (100 ng) was subjected to sequencing library construction using a TruSeq RNA Access Library Prep Kit (Illumina) according to the manufacturer’s protocols. The total RNA was first fragmented into small pieces using divalent cations at elevated temperatures. The cleaved RNA fragments were copied into first-strand cDNA using SuperScript II reverse transcriptase (Invitrogen) and random primers. This was followed by second strand cDNA synthesis using DNA polymerase I, RNase H, and dUTP. These cDNA fragments then go through an end repair process, the addition of a single ‘A’ base, and then ligation of the adapters. The products were then purified and enriched by PCR to create a cDNA library. All libraries were normalized, and six libraries were pooled into a single hybridization/capture reaction. Pooled libraries were incubated with a cocktail of biotinylated oligonucleotides corresponding to the coding regions of the genome. The targeted library molecules were captured via hybridized biotinylated oligo probes using streptavidin-conjugated beads. After two rounds of hybridization/capture reactions, the enriched library molecules were subjected to a second round of PCR amplification***. ***Captured libraries were quantified using KAPA Library Quantification kits for Illumina Sequencing platforms according to the qPCR Quantification Protocol Guide (KAPA BIOSYSTEMS) and qualified using the TapeStation D1000 ScreenTape (Agilent Technologies). Indexed libraries were then submitted to an Illumina HiSeq 2500 (Illumina, Inc.), and paired-end (2 × 100 bp) sequencing was performed by Macrogen Inc.

### MiRNA-based gene ontology (GO) analysis of the target genes

The web-based computational tool DIANA-miRPath v3.0, was used for the GO analysis of the interaction of the genes identified with hsa-mir-17-5p, hsa-mir-183-5p, and hsa-mir-20a-5p. The *p*-value threshold was set to 0.05.

### Statistical analysis

The GraphPad Prism 9 software was used for all analyses. The results are shown as individual dot plots for at least three independent experiments. A two-way ANOVA was used for all comparisons.

## Supplementary Information

**Additional file 1: Figure S1. **Protective effect of F3, human neural stem cells on 6-OHDA treated SH-SY5Y cells. To determine the optimal dose for 6-OHDA neurotoxin-induced cell death, SH-SY5Y cells (human neuroblastoma cells showing dopaminergic neuron-like phenotype) were treated with 150, 250, 350, and 450 µM 6-OHDA. (A) Cell viability was measured using the CCK-8 assay 24 h after 6-OHDA treatment. Cell viability was almost 50 % when cells were treated with 450 µM 6-OHDA. (B) Live/dead cell population in 6-OHDA-treated SH-SY5Y cells and cells co-treated with F3 cells were determined by the live/dead assay. In the F3-treated group, cell death by 6-OHDA was reduced. **Figure S2. **Characterization of isolated EVs derived from HFF and F3 cells. (A) Representative analysis results of EV markers were shown (1. Tetraspanins-CD9, CD63, CD81; 2. Cytosolic proteins-TSG101; Absent of intracellular protein in EVs-Calnexin). As a control, actin was tested by immunoblot on the same samples. (B, C) NanoSight representative images of HFF (B) and F3 cell derived EV samples (C) showed the size of particles around 80-150 nm. **Figure S3. **ROS-induced apoptotic pathway-related toxic effects of 6-OHDA on SH-SY5Y cells. SH-SY5Y cells were treated with 150, 250, 350, and 450 µM 6-OHDA. (A) To measure intracellular ROS, the fluorescence intensity of DCFDA was measured. (B) Mitochondrial membrane potential (MMP) levels were analyzed by JC-1 dye staining. (C) Caspase 3/7 activity was measured using the Caspase-Glo 3/7 reagent. (D) The late apoptotic cell population was confirmed by flow cytometry in SH-SY5Y cells after PI staining. The labeled percentages indicate the late apoptotic cell populations. Cell death, ROS generation, MMP changes, and caspase 3/7 cleavage were increased in a 6-OHDA dose-dependent manner. (E) JC-1 dye was used to measure mitochondrial membrane potential (MMP), and the ratio of red/green fluorescence intensity was analyzed. (F) The late apoptotic population was confirmed by flow cytometry after PI staining. The labeled percentage indicates late apoptotic cell populations. F3-derived EVs reduced 6-OHDA-induced late apoptotic SH-SY5Y cells. **Figure S4. **Anti-inflammatory effect of F3-derived EVs in LPS-treated BV2 cells*. *(A-B) After BV2 microglia cells were treated with 0.01, 0.05, 0.1, 0.5, 1, and 5 µg/mL LPS, the RNA levels of *IL-1α* and *IL-1β* were measured by PCR and qPCR. (C-E) After treatment of 0.01 µg/mL LPS to BV2 cells pre-treated with F3-derived EVs, the RNA expression levels of *IL-1α* and *IL-1β* were measured. *IL-1α and* *IL-1β *level in LPS-treated BV2 cells were less increased after treatment of F3- or HFF-derived EVs. (F) TNF-α production was measured by ELISA assay after treatment of LPS to BV2 cells pre-treated with F3- or HFF-derived EVs. The level of released TNF-α was attenuated after treatment with F3-derived EVs. **Figure S5. **The bioanalyzer results of exosomal RNAs isolated from HFF- and F3-derived EVs. Exosomal RNA was isolated from HFF and F3 cell derived EVs and their quality was analyzed with (A) Bioanalyzer RNA Pico 6000 chip and (B) Bioanalyzer RNA Small RNA chip. **Figure S6. **The different long non-coding RNA (lncRNA) expression profiles between F3- and HFF-derived EVs by small RNA sequencing. (A) The heatmap revealed the distinct lncRNA expression profiles between F3- and HFF-derived EVs. The criteria was more than two-fold changes and the value 8 of normalized log2 data (total 109 lncRNAs). (B) Venn diagram of EV lncRNA differentially expressed in F3- versus HFF-derived EVs. The criteria were more than the value 8 of normalized log2 data. The top 10 lncRNA from uniquely expressed in F3-derived EVs were listed. (C) The scatter plot measuring lncRNA expression by comparing F3- and HFF-derived EVs. The criteria were a two-fold difference of log2. (Red dots, high relative expression; green dots, low relative expression). (D) Pie chart of the percentages indicates a significant increase in lncRNA on F3-derived EVs compared to HFF-derived EVs among total lncRNA associated with gene categories. The criteria were more than two-fold changes and the value 4 of normalized log2 data. (E) The bar graph of the percentage of up and down significant lncRNA was shown based on the gene categories. The criteria were more than two-fold changes and the value 4 of normalized log2 data. (F) The expression plot of normalized log2 data in neurogenesis and cell differentiation categories was shown. (G) Expression plot of normalized log2 data in immune and inflammatory response categories was shown. **Figure S7. **Characteristics of EVs derived from F3 or F3-palm-tdTomato cells. (A) F3-palm-tdTomato cells were imaged using confocal microscopy on day 1, 3, and 7 after FACS sorting of tdTomato-positive cells. Scale bar = 20 µm (B) The fluorescence intensity of tdTomato-infected cells was measured on day 1, 3, and 7. The fluorescence intensity of the sorted F3-palm-tdTomato cells were maintained for a week. (C) The size of EVs before and after palm-tdTomato virus infection was measured using Nanosight size detection system. (D) The fluorescence intensity of EVs isolated from F3 and F3-palm-tdTomato cells was measured. (E) The markers of EVs such as CD63 or CD81 were evaluated on F3- and F3-palm-tdTomato-derived EVs using western blotting. Characteristics of cell-derived EVs were maintained even after palm-tdTomato infection. (F) In order to check the time for EV uptake in SH-SY5Y cells, the isolated F3-palm-tdTomato cell-derived EVs were treated. TdTomato fluorescence signals were identified inside the cells using confocal microscopy. **Figure S8. In vivo** F3 or F3-EVs tracing using luciferase reporter- or a Palm-tdTomato-expressing F3 stable cell line. (A) F3 cells were infected with EF1a-luc2-GFP lentivirus and GFP-positive cells (arrows) were observed under a confocal microscope. Scale bar = 100 µm. (B) The luciferase activities were measured in F3-EF1a-luc2-GFP cells. (C) Bioluminescence imaging was obtained 1, 3 and 6 days after injection of F3-EF1a-luc2-GFP cells into SN of normal mouse brain. F3 cells injected into SN were maintained *in vivo* for a week. For each day, three mice were used for imaging. (D) Brain cross-sectional images were obtained at 24 h after injection of F3-palm-tdTomato cells into SN of normal mouse brain. In the SN region, the tdTomato fluorescence signals at the injection site (indicated as 1.) and adjacent to the injection site (indicated as 2.) were analyzed via z-stack imaging. (E) Magnified images of F3-palm-tdTomato cells were obtained in the region adjacent to the injection site using a super resolution microscopy (stochastic optical reconstruction microscopy; STORM). Scale bar = 1 µm. Vesicle-like tdTomato fluorescence signals were detected at the membrane area of cells (indicated with arrows). (F) The 3D transparent mouse brain was created before (left) and after (right) a process called CLARITY. The red arrow is the injection site. The enlarged images of injected sites are shown. Scale bar = 100 µm. **Figure S9. **Establishment of 6-OHDA induced PD mouse model. After one week of intracerebral injection of 6-OHDA into mice, the pathology was evaluated. (A) Apomorphine-induced rotation behavior tests of mice injected with PBS or 6-OHDA were analyzed. (B) TH immunofluorescence staining was performed to detect the expression of TH-positive dopaminergic neurons in the striatum. The 6-OHDA-induced PD model showed dopaminergic neuronal loss and impaired motor function. Scale bar = 100 µm.

**Additional file 2.** List of microRNAs and long noncoding RNAs identified from small RNA sequencing of F3 cell- and HFF cell-derived EVs.

## Data Availability

The authors declare that all data supporting the findings of this study are available within the manuscript or are available from the corresponding authors upon request.

## Abbreviations

NSCs: Neural stem cells
PD: Parkinson’s disease
EVs: Extracellular vesicles
6-OHDA: 6-Hydroxydopamine
ROS: Reactive oxygen species
miRNAs: MicroRNAs
SN: Substantia nigra
PET: Positron emission tomography
Th17: T helper 17
F3: F3 cell
CCK-8: Cell counting kit-8
DCF-DA: 2,7-Dichlorofluorescein diacetate
PDMS: Polydimethysiloxane
qPCR: Quantitative PCR
AP: Anterioposterior
ML: Mediolateral
DV: Dorsoventral
TBS: Tris-buffered saline
GFAP: Glial fibrillary acidic protein
GO: Gene ontology
IL-1α: Interleukin-1 alpha
IL-1β: Interleukin-1 beta
TNF-α: Tumor necrosis factor-alpha
LPS: Lipopolysaccharide
lncRNAs: Long noncoding RNAs
iPSCs: Induced pluripotent stem cells
TRK: Tropomyosin receptor kinase
EF1a-luc2-GFP: Elongation factor 1 alpha promotor-driven luciferase 2-green fluorescent protein
Palm-tdTomato: Palmitoylated-tdTomato
CNS: Central nervous system
MSCs: Mesenchymal stem cells
HFF: Human foreskin fibroblasts
DMEM: Dulbecco’s modified Eagle’s medium
FBS: Fetal bovine serum
EthD-1: Ethidium homodimer
DPBS: Dulbecco’s phosphate-buffered saline
FACS: Fluorescence-activated cell sorting
TFF: Tangential flow filtration
NTA: Nanoparticle tracking analysis
MMP: Mitochondrial membrane potential
STORM: Stochastic optical reconstruction microscopy
