# Supplementary material for: Human neural stem cell-derived extracellular vesicles protect against Parkinson’s disease pathologies
Source: J Nanobiotechnology. 2022 Apr 25;20:198. doi: 10.1186/s12951-022-01356-2 (PMC9040239; doi:10.1186/s12951-022-01356-2)
Supplement: Supplementary file 1 — Additional file 1: Figure S1. Protective effect of F3, human neural stem cells on 6-OHDA treated SH-SY5Y cells. To determine the optimal dose for 6-OHDA neurotoxin-induced cell death, SH-SY5Y cells (human neuroblastoma cells showing dopaminergic neuron-like phenotype) were treated with 150, 250, 350, and 450 µM 6-OHDA. (A) Cell viability was measured using the CCK-8 assay 24 h after 6-OHDA treatment. Cell viability was almost 50 % when cells were treated with 450 µM 6-OHDA. (B) Live/dead cell population in 6-OHDA-treated SH-SY5Y cells and cells co-treated with F3 cells were determined by the live/dead assay. In the F3-treated group, cell death by 6-OHDA was reduced. Figure S2. Characterization of isolated EVs derived from HFF and F3 cells. (A) Representative analysis results of EV markers were shown (1. Tetraspanins-CD9, CD63, CD81; 2. Cytosolic proteins-TSG101; Absent of intracellular protein in EVs-Calnexin). As a control, actin was tested by immunoblot on the same samples. (B, C) NanoSight representative images of HFF (B) and F3 cell derived EV samples (C) showed the size of particles around 80-150 nm. Figure S3. ROS-induced apoptotic pathway-related toxic effects of 6-OHDA on SH-SY5Y cells. SH-SY5Y cells were treated with 150, 250, 350, and 450 µM 6-OHDA. (A) To measure intracellular ROS, the fluorescence intensity of DCFDA was measured. (B) Mitochondrial membrane potential (MMP) levels were analyzed by JC-1 dye staining. (C) Caspase 3/7 activity was measured using the Caspase-Glo 3/7 reagent. (D) The late apoptotic cell population was confirmed by flow cytometry in SH-SY5Y cells after PI staining. The labeled percentages indicate the late apoptotic cell populations. Cell death, ROS generation, MMP changes, and caspase 3/7 cleavage were increased in a 6-OHDA dose-dependent manner. (E) JC-1 dye was used to measure mitochondrial membrane potential (MMP), and the ratio of red/green fluorescence intensity was analyzed. (F) The late apoptotic population was confirmed [file 12951_2022_1356_MOESM1_ESM.docx]

**Human Neural Stem Cell-Derived Extracellular Vesicles Protect Against Parkinson’s disease Pathologies**

Eun Ji Lee^1,2,3^, Yoori Choi^1,2,3^, Hong J Lee^4,5^, Do Won Hwang^1,6,*^, Dong Soo Lee^1,2,3,*^

^1^Department of Nuclear Medicine, Seoul National University College of Medicine, Korea; ^2^Department of Nuclear Medicine, Seoul National University Hospital, Seoul, Korea; ^3^Department of Molecular Medicine and Biopharmaceutical Sciences, Graduate School of Convergence Science and Technology, Seoul National University, Seoul, Korea; ^4^College of Medicine and Medical Research Institute, Chungbuk National University, Cheongju, Chungbuk, Korea; ^5^Research Institute, huMetaCELL Inc., 220, Bugwang-ro, Bucheon-si, Gyeonggi-do, Republic of Korea; ^6^THERABEST, Inc. Seocho-daero 40-gil, Seoul, 06657, Korea

**FIGURE LEGENDS**


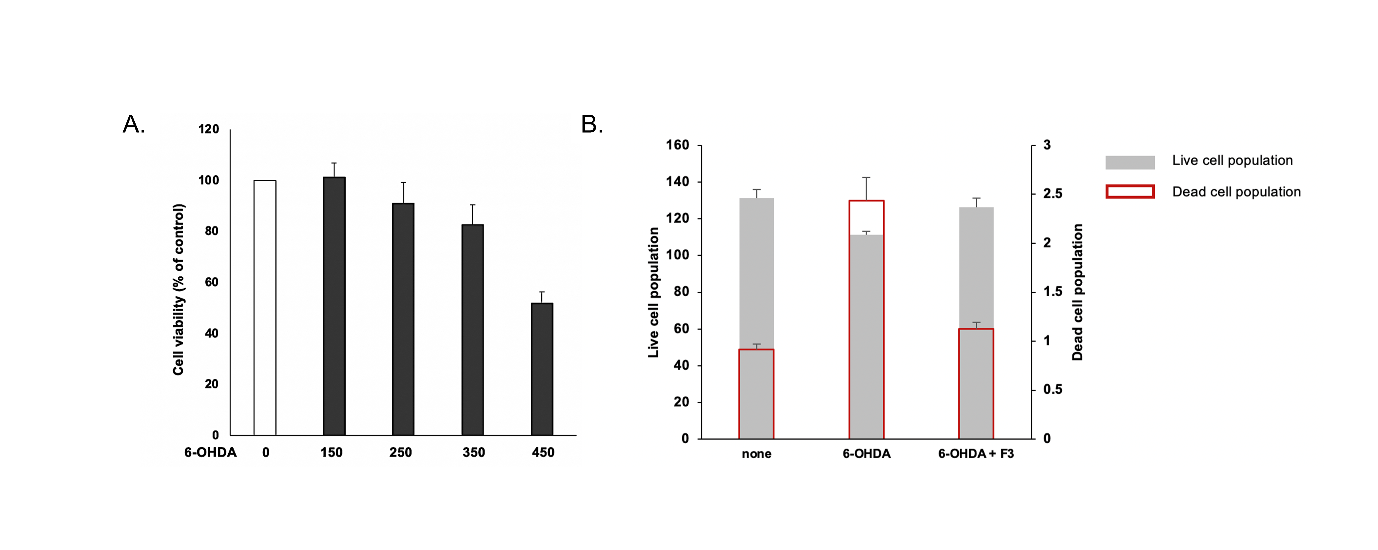


**Figure S1. Protective effect of F3, human neural stem cells on 6-OHDA treated SH-SY5Y cells.** To determine the optimal dose for 6-OHDA neurotoxin-induced cell death, SH-SY5Y cells (human neuroblastoma cells showing dopaminergic neuron-like phenotype) were treated with 150, 250, 350, and 450 µM 6-OHDA. (A) Cell viability was measured using the CCK-8 assay 24 h after 6-OHDA treatment. Cell viability was almost 50 % when cells were treated with 450 µM 6-OHDA. (B) Live/dead cell population in 6-OHDA-treated SH-SY5Y cells and cells co-treated with F3 cells were determined by the live/dead assay. In the F3-treated group, cell death by 6-OHDA was reduced.

**
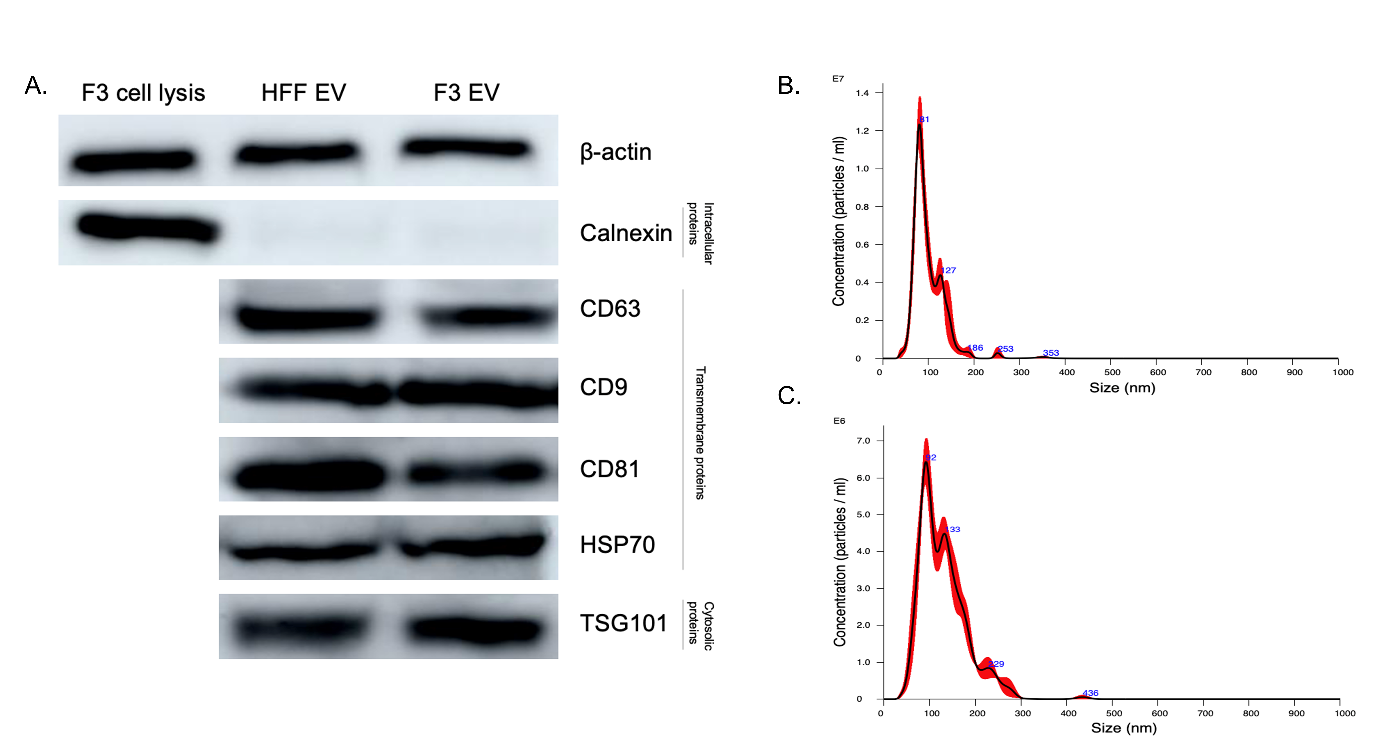
**

**Figure S2. Characterization of isolated EVs derived from HFF and F3 cells.** (A) Representative analysis results of EV markers were shown (1. Tetraspanins-CD9, CD63, CD81; 2. Cytosolic proteins-TSG101; Absent of intracellular protein in EVs-Calnexin). As a control, actin was tested by immunoblot on the same samples. (B, C) NanoSight representative images of HFF (B) and F3 cell derived EV samples (C) showed the size of particles around 80-150 nm.

**
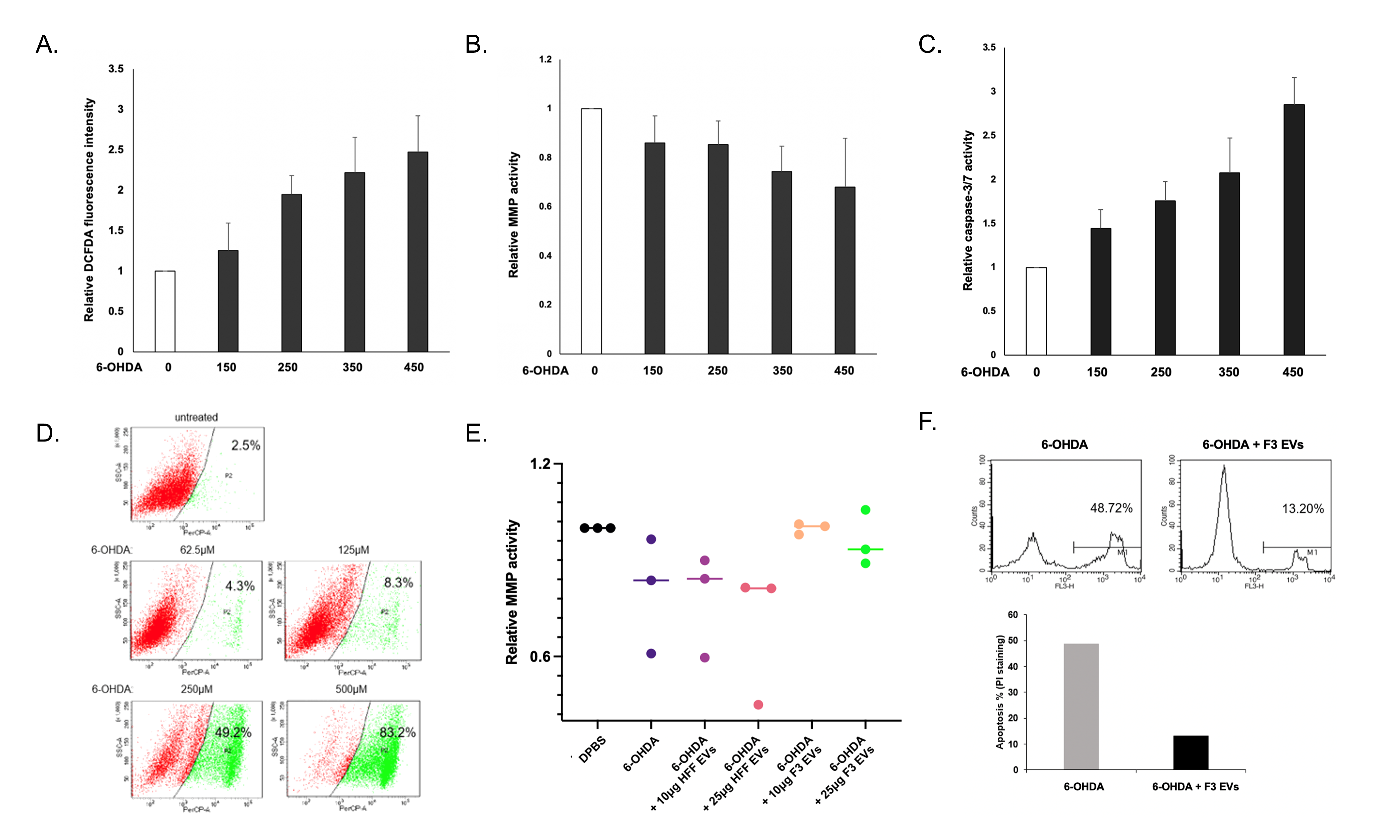
**

**Figure S3. ROS-induced apoptotic pathway-related toxic effects of 6-OHDA on SH-SY5Y cells.** SH-SY5Y cells were treated with 150, 250, 350, and 450 µM 6-OHDA. (A) To measure intracellular ROS, the fluorescence intensity of DCFDA was measured. (B) Mitochondrial membrane potential (MMP) levels were analyzed by JC-1 dye staining. (C) Caspase 3/7 activity was measured using the Caspase-Glo 3/7 reagent. (D) The late apoptotic cell population was confirmed by flow cytometry in SH-SY5Y cells after PI staining. The labeled percentages indicate the late apoptotic cell populations. Cell death, ROS generation, MMP changes, and caspase 3/7 cleavage were increased in a 6-OHDA dose-dependent manner. (E) JC-1 dye was used to measure mitochondrial membrane potential (MMP), and the ratio of red/green fluorescence intensity was analyzed. (F) The late apoptotic population was confirmed by flow cytometry after PI staining. The labeled percentage indicates late apoptotic cell populations. F3-derived EVs reduced 6-OHDA-induced late apoptotic SH-SY5Y cells.

**
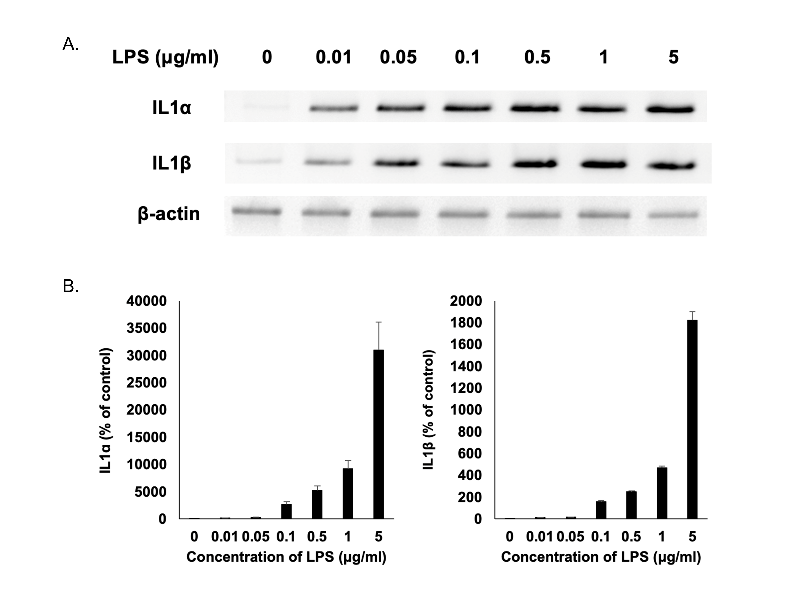
**

**
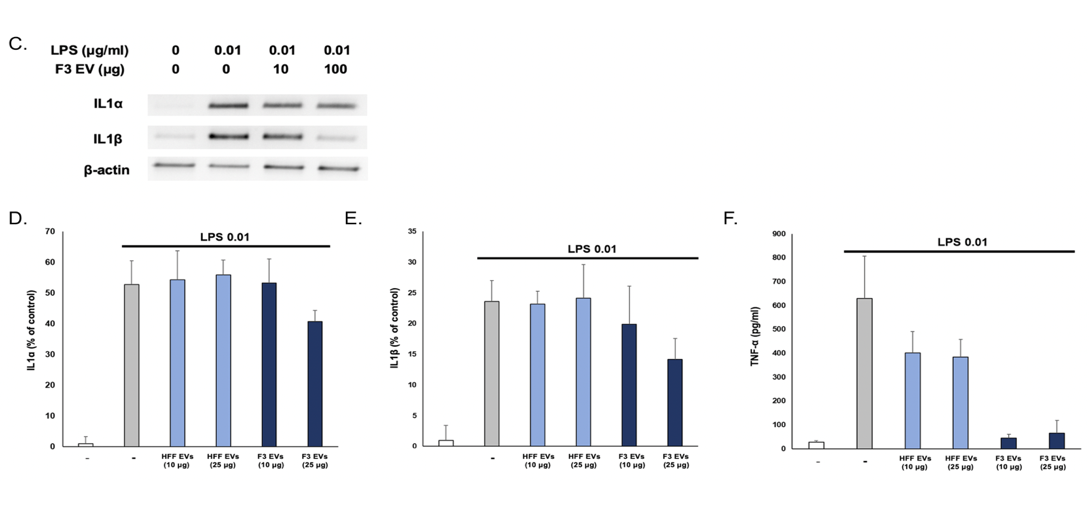
**

**Figure S4. Anti-inflammatory effect of F3-derived EVs in LPS-treated BV2 cells*.*** (A-B) After BV2 microglia cells were treated with 0.01, 0.05, 0.1, 0.5, 1, and 5 µg/mL LPS, the RNA levels of *IL-1α* and *IL-1β* were measured by PCR and qPCR. (C-E) After treatment of 0.01 µg/mL LPS to BV2 cells pre-treated with F3-derived EVs, the RNA expression levels of *IL-1α* and *IL-1β* were measured. *IL-1α and* *IL-1β* level in LPS-treated BV2 cells were less increased after treatment of F3- or HFF-derived EVs. (F) TNF-α production was measured by ELISA assay after treatment of LPS to BV2 cells pre-treated with F3- or HFF-derived EVs. The level of released TNF-α was attenuated after treatment with F3-derived EVs.

**
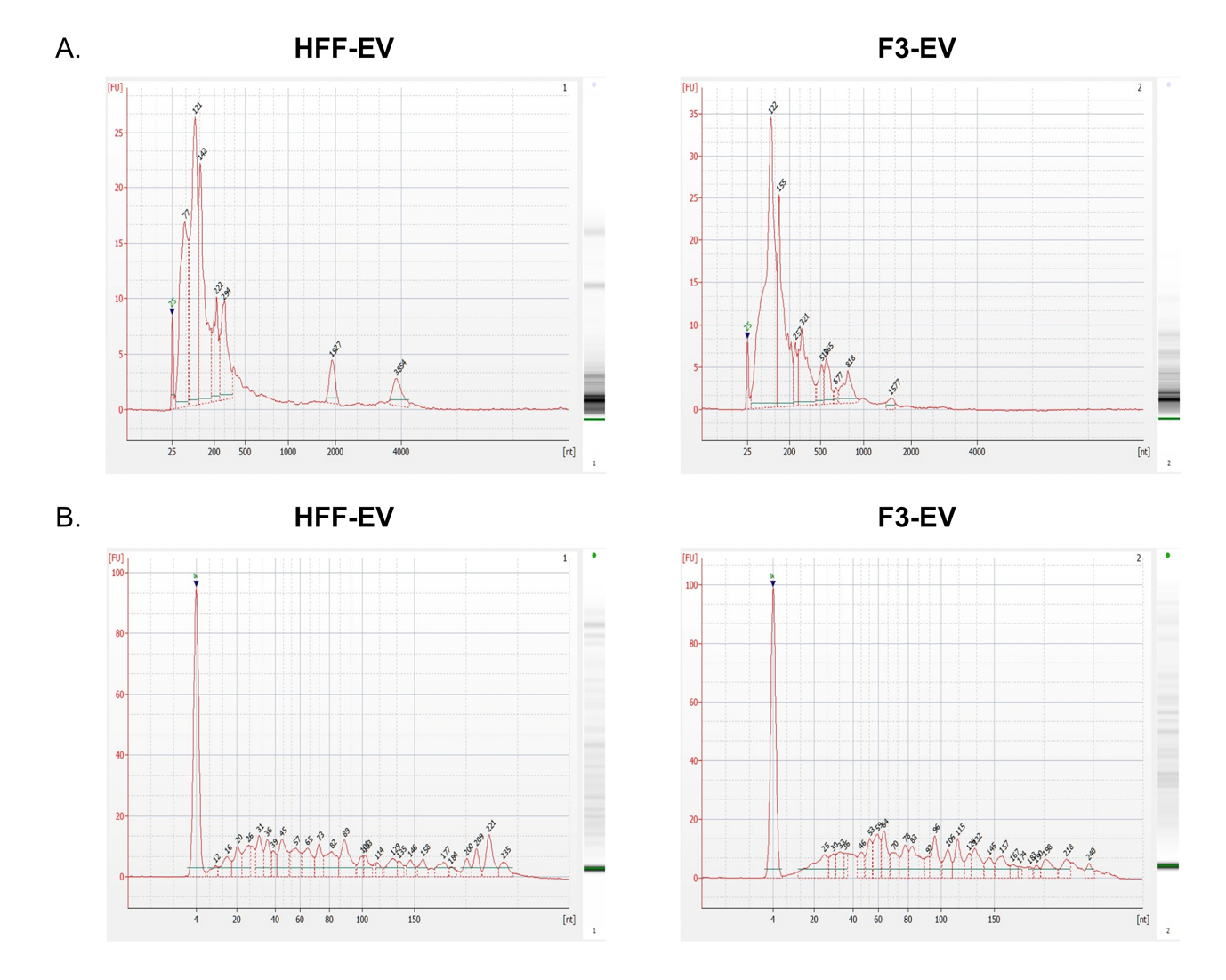
**

**Figure S5. The bioanalyzer results of exosomal RNAs isolated from HFF- and F3-derived EVs.** Exosomal RNA was isolated from HFF and F3 cell derived EVs and their quality was analyzed with (A) Bioanalyzer RNA Pico 6000 chip and (B) Bioanalyzer RNA Small RNA chip.

**
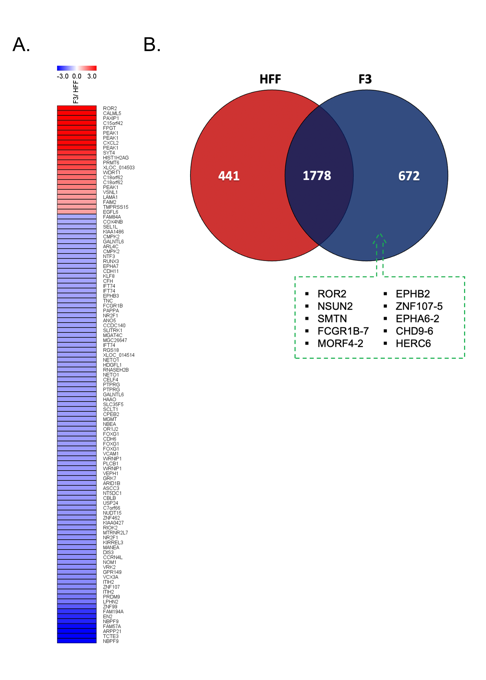

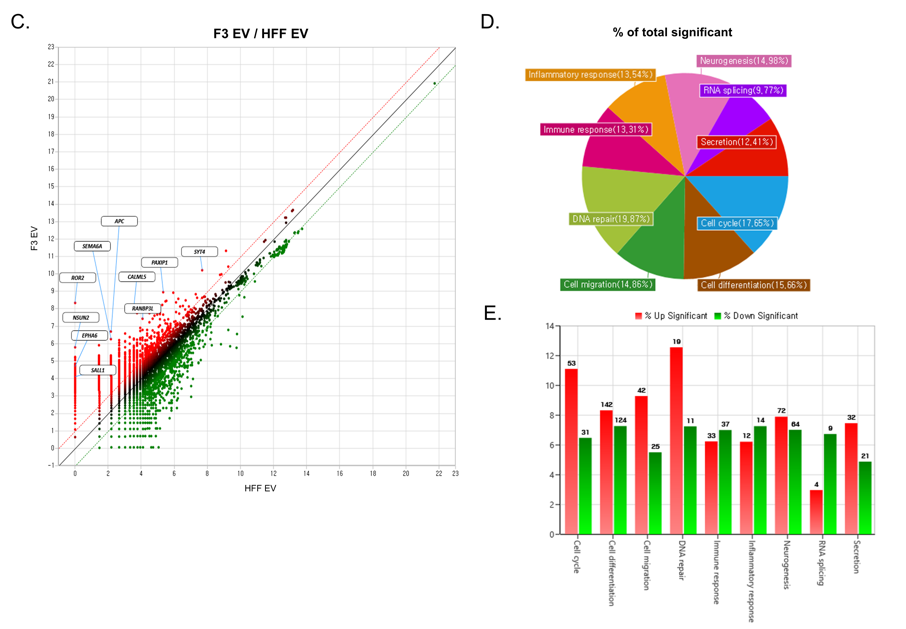
**

**
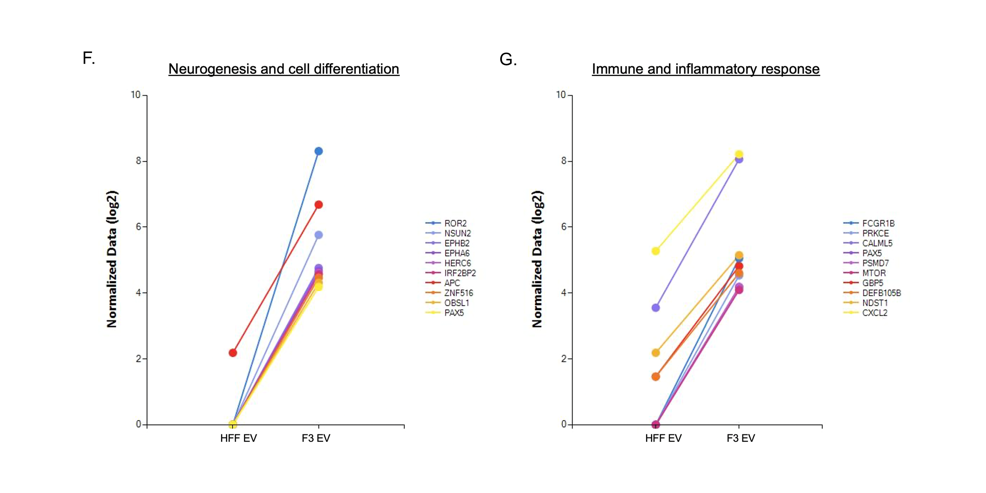
**

**Figure S6. The different long non-coding RNA (lncRNA) expression profiles between F3- and HFF-derived EVs by small RNA sequencing.** (A) The heatmap revealed the distinct lncRNA expression profiles between F3- and HFF-derived EVs. The criteria was more than two-fold changes and the value 8 of normalized log2 data (total 109 lncRNAs). (B) Venn diagram of EV lncRNA differentially expressed in F3- versus HFF-derived EVs. The criteria were more than the value 8 of normalized log2 data. The top 10 lncRNA from uniquely expressed in F3-derived EVs were listed. (C) The scatter plot measuring lncRNA expression by comparing F3- and HFF-derived EVs. The criteria were a two-fold difference of log2. (Red dots, high relative expression; green dots, low relative expression). (D) Pie chart of the percentages indicates a significant increase in lncRNA on F3-derived EVs compared to HFF-derived EVs among total lncRNA associated with gene categories. The criteria were more than two-fold changes and the value 4 of normalized log2 data. (E) The bar graph of the percentage of up and down significant lncRNA was shown based on the gene categories. The criteria were more than two-fold changes and the value 4 of normalized log2 data. (F) The expression plot of normalized log2 data in neurogenesis and cell differentiation categories was shown. (G) Expression plot of normalized log2 data in immune and inflammatory response categories was shown.

**
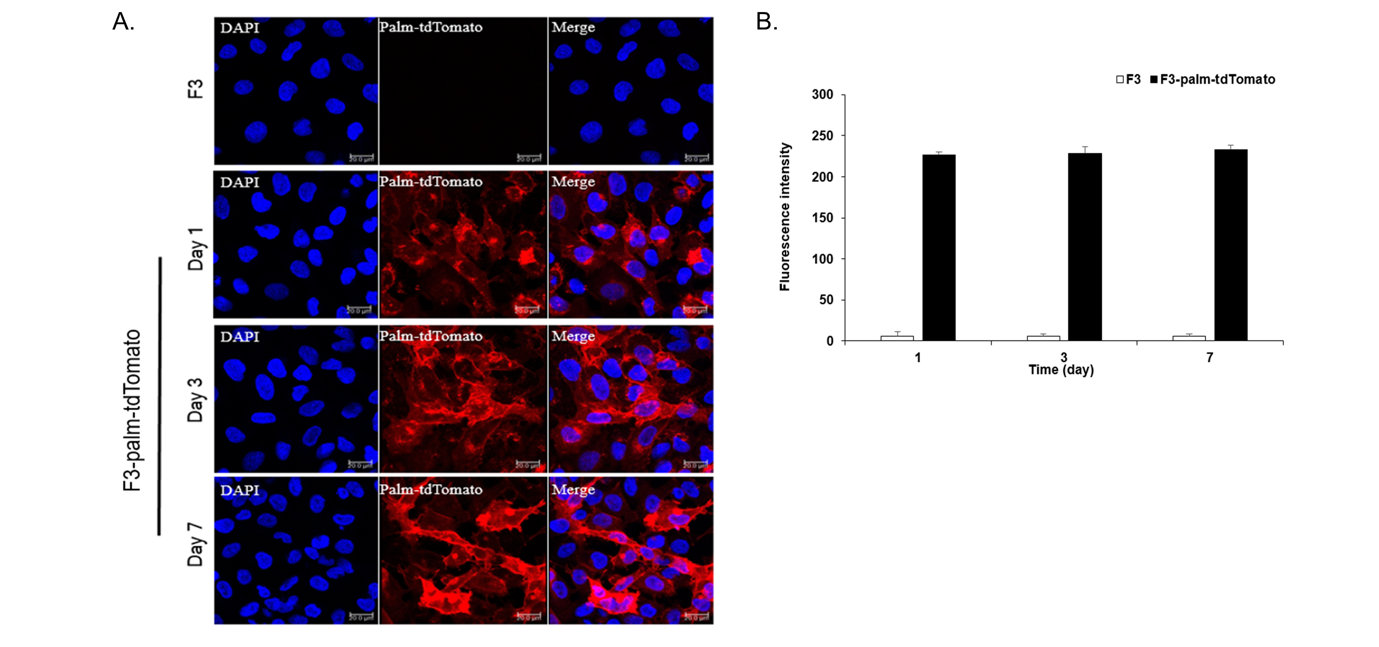
**

**
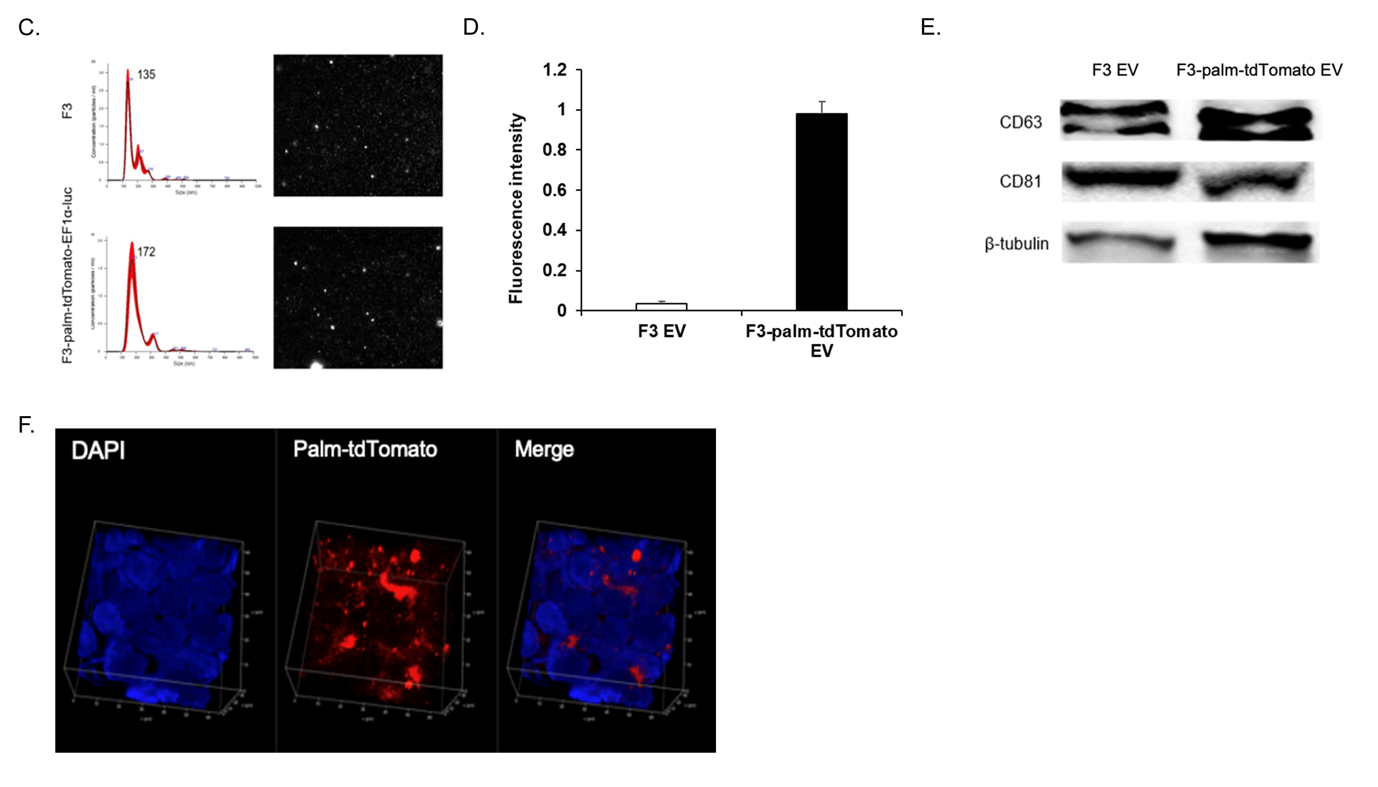
**

**Figure S7. Characteristics of EVs derived from F3 or F3-palm-tdTomato cells.** (A) F3-palm-tdTomato cells were imaged using confocal microscopy on day 1, 3, and 7 after FACS sorting of tdTomato-positive cells. Scale bar = 20 µm (B) The fluorescence intensity of tdTomato-infected cells was measured on day 1, 3, and 7. The fluorescence intensity of the sorted F3-palm-tdTomato cells were maintained for a week. (C) The size of EVs before and after palm-tdTomato virus infection was measured using Nanosight size detection system. (D) The fluorescence intensity of EVs isolated from F3 and F3-palm-tdTomato cells was measured. (E) The markers of EVs such as CD63 or CD81 were evaluated on F3- and F3-palm-tdTomato-derived EVs using western blotting. Characteristics of cell-derived EVs were maintained even after palm-tdTomato infection. (F) In order to check the time for EV uptake in SH-SY5Y cells, the isolated F3-palm-tdTomato cell-derived EVs were treated. TdTomato fluorescence signals were identified inside the cells using confocal microscopy.

**
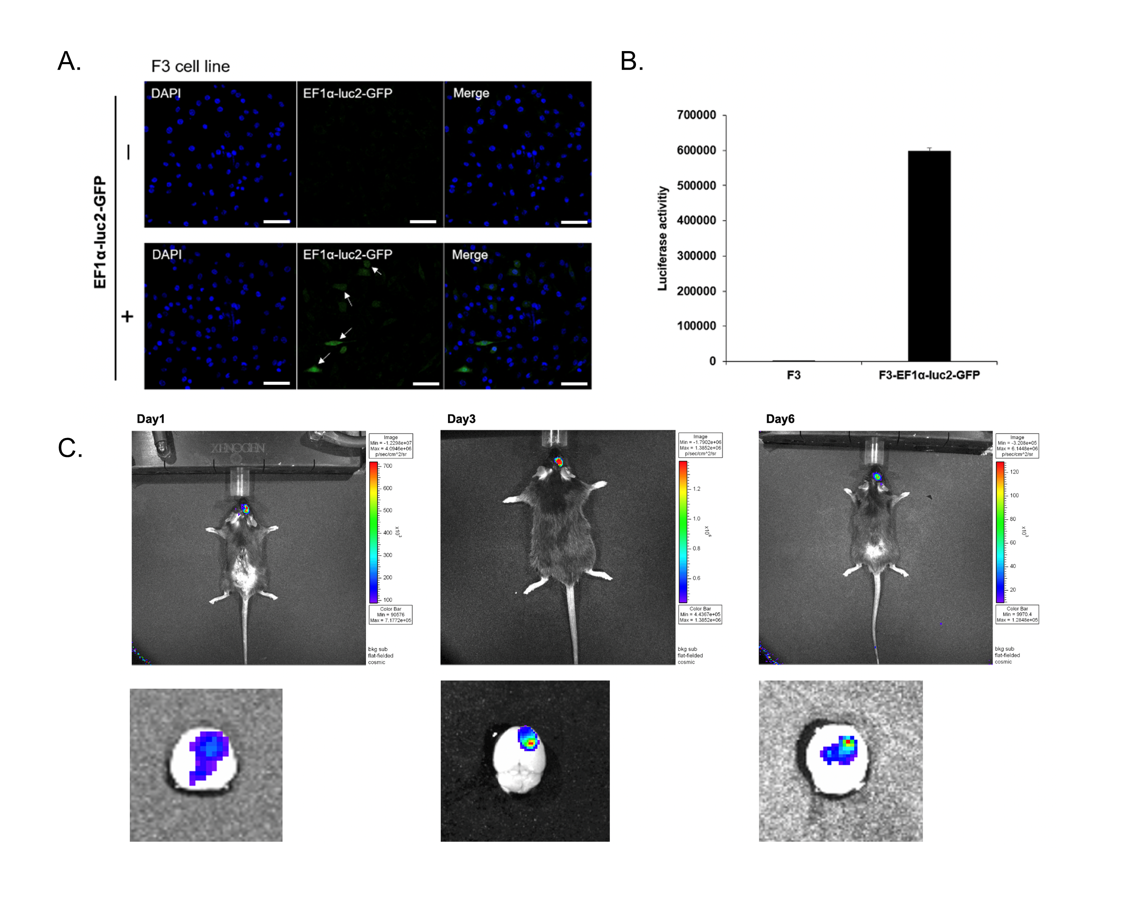
**

**
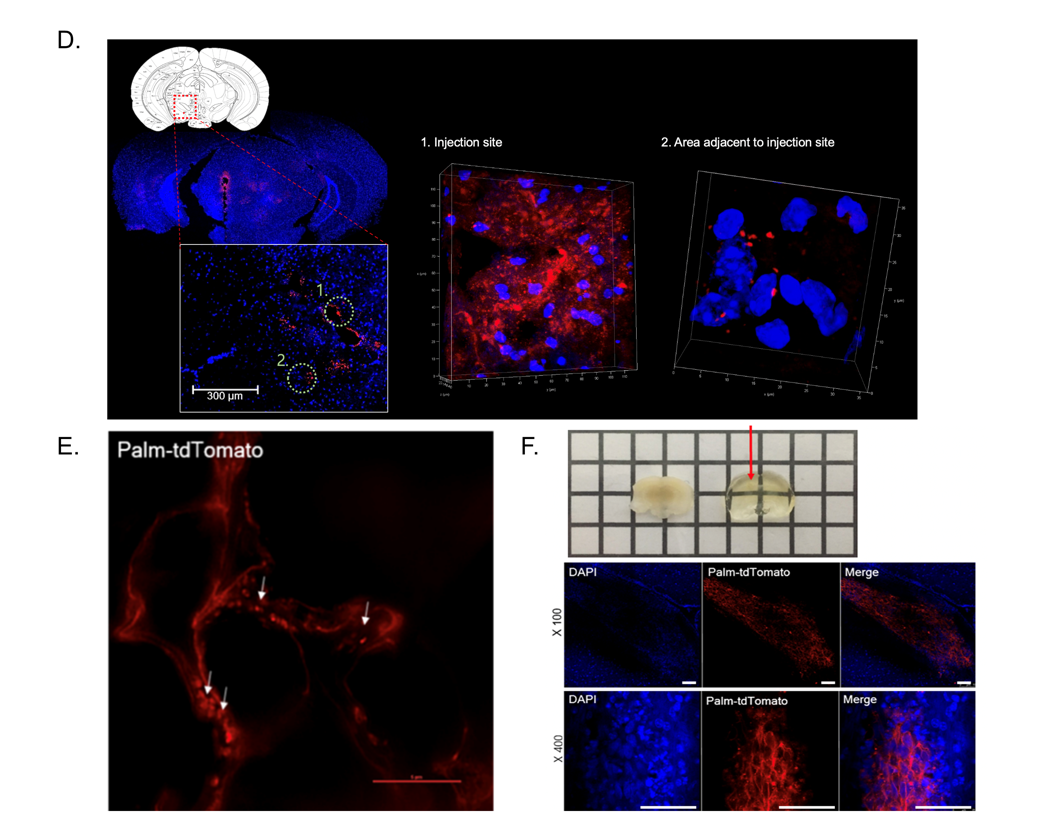
**

**Figure S8. *In vivo* F3 or F3-EVs tracing using luciferase reporter- or a Palm-tdTomato-expressing F3 stable cell line.** (A) F3 cells were infected with EF1a-luc2-GFP lentivirus and GFP-positive cells (arrows) were observed under a confocal microscope. Scale bar = 100 µm. (B) The luciferase activities were measured in F3-EF1a-luc2-GFP cells. (C) Bioluminescence imaging was obtained 1, 3 and 6 days after injection of F3-EF1a-luc2-GFP cells into SN of normal mouse brain. F3 cells injected into SN were maintained *in vivo* for a week. For each day, three mice were used for imaging. (D) Brain cross-sectional images were obtained at 24 h after injection of F3-palm-tdTomato cells into SN of normal mouse brain. In the SN region, the tdTomato fluorescence signals at the injection site (indicated as 1.) and adjacent to the injection site (indicated as 2.) were analyzed via z-stack imaging. (E) Magnified images of F3-palm-tdTomato cells were obtained in the region adjacent to the injection site using a super resolution microscopy (stochastic optical reconstruction microscopy; STORM). Scale bar = 1 µm. Vesicle-like tdTomato fluorescence signals were detected at the membrane area of cells (indicated with arrows). (F) The 3D transparent mouse brain was created before (left) and after (right) a process called CLARITY. The red arrow is the injection site. The enlarged images of injected sites are shown. Scale bar = 100 µm.

**
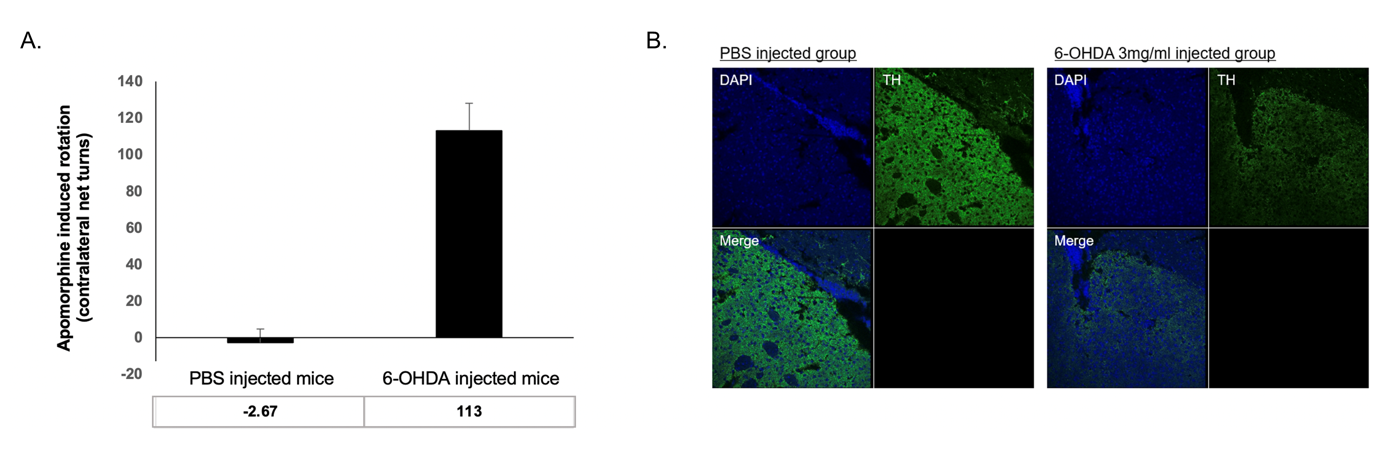
**

**Figure S9. Establishment of 6-OHDA induced PD mouse model.** After one week of intracerebral injection of 6-OHDA into mice, the pathology was evaluated. (A) Apomorphine-induced rotation behavior tests of mice injected with PBS or 6-OHDA were analyzed. (B) TH immunofluorescence staining was performed to detect the expression of TH-positive dopaminergic neurons in the striatum. The 6-OHDA-induced PD model showed dopaminergic neuronal loss and impaired motor function. Scale bar = 100 µm.
